# After the Honeymoon: Neural and Genetic Correlates of Romantic Love in Newlywed Marriages

**DOI:** 10.3389/fpsyg.2020.00634

**Published:** 2020-05-07

**Authors:** Bianca P. Acevedo, Michael J. Poulin, Nancy L. Collins, Lucy L. Brown

**Affiliations:** ^1^Neuroscience Research Institute and Department of Psychological and Brain Sciences, University of California, Santa Barbara, Santa Barbara, CA, United States; ^2^Department of Psychology, University at Buffalo, Buffalo, NY, United States; ^3^Department of Neurology, Albert Einstein College of Medicine, Bronx, NY, United States

**Keywords:** fMRI, marriage, dopamine, romantic love, pair-bonds

## Abstract

In Western culture, romantic love is commonly a basis for marriage. Although it is associated with relationship satisfaction, stability, and individual well-being, many couples experience declines in romantic love. In newlyweds, specifically, changes in love predict marital outcomes. However, the biological mechanisms underlying the critical transition to marriage are unknown. Thus, for the first time, we explored the neural and genetic correlates of romantic love in newlyweds. Nineteen first-time newlyweds were scanned (with functional MRI) while viewing face images of the partner versus a familiar acquaintance, around the time of the wedding (T1) and 1 year after (T2). They also provided saliva samples for genetic analysis (*AVPR1a* rs3, *OXTR* rs53576, *COMT* rs4680, and *DRD4*-7R), and completed self-report measures of relationship quality including the Eros (romantic love) scale. We hypothesized that romantic love is a developed form of the mammalian drive to find, and keep, preferred mates; and that its maintenance is orchestrated by the brain’s reward system. Results showed that, at both time points, romantic love maintenance (Eros difference score: T2-T1) was associated with activation of the dopamine-rich substantia nigra in response to face images of the partner. Interactions with vasopressin, oxytocin, and dopamine genes implicated in pair-bonding (*AVPR1a* rs3, *OXTR* rs53576, *COMT* rs4680, and *DRD4*-7R) also conferred strong activation in the dopamine-rich ventral tegmental area at both time points. Consistent with work highlighting the role of sexual intimacy in relationships, romantic love maintenance showed correlations in the paracentral lobule (genital region) and cortical areas involved in sensory and cognitive processing (occipital, angular gyrus, insular cortex). These findings suggest that romantic love, and its maintenance, are orchestrated by dopamine-, vasopressin- and oxytocin-rich brain regions, as seen in humans and other monogamous animals. We also provide genetic evidence of polymorphisms associated with oxytocin, vasopressin and dopamine function that affect the propensity to sustain romantic love in early stage marriages. We conclude that romantic love maintenance is part of a broad mammalian strategy for reproduction and long-term attachment that is influenced by basic reward circuitry, complex cognitive processes, and genetic factors.

## Introduction

Pair-bonds appear in nearly all human societies (Schacht and Kramer, 2019), and across other mammalian species (Walum and Young, 2018). Monogamous pair-bonds are characterized by selective partner preference, cohabitation, bi-parental care of offspring, aggression toward strangers, and coordinated behaviors between the couple (Getz et al., 1981; Mendoza and Mason, 1986; de Waal and Gavrilets, 2013; Lukas and Clutton-Brock, 2013). Pair-bonds are thought to have evolved to increase the survival and success of offspring (Walum and Young, 2018). In recent decades ideas about their function have expanded to include companionship, care, and evolutionary fitness (de Waal, 2008; Batson, 2011; Raghanti et al., 2018).

In Western culture, romantic love—defined as a drive for union with a specific other that involves excitement, engagement, and sexual desire (Berscheid and Hatfield, 1969; Acevedo and Aron, 2009)—is closely intertwined with marriage. Romantic love is associated with relationship satisfaction and stability, and individuals’ health and well-being. However, for many couples it fades over time (Hatfield et al., 2008). Declines in romantic love often signal trouble for couples, as they are correlated with marital dissatisfaction, increased attention to alternative partners, extra-marital affairs, and divorce (Huston and Houts, 1998; Miller et al., 2006; Maner et al., 2009; Nowak et al., 2014). In addition, relationship problems are non-trivially associated with higher rates of mental and physical health problems, suicide, and homicide (Levenson et al., 1993). Thus, it is important to understand what may help couples to sustain romantic love to ensure the success of marriages and the family unit.

The newlywed years are a critical time that predict long-term marital outcomes (Miller et al., 2006). Specifically, researchers have described “honeymoon effects” in which initially positive and romantic marriages turn sour over time, with sharp declines in love, affection, and positive affect (McNulty et al., 2013; Lorber et al., 2015). Several explanations have been offered for declines in love, from cognitively and perceptually focused “disillusionment” models (Huston et al., 2001; van Dijk and Zeelenberg, 2002; Niehuis et al., 2011, 2018), to affectively focused models centering on increases in stress, negative emotions, and conflict (Bradbury, 1998; Gottman et al., 1998). Yet other models have suggested that habituation, the flattening out of intimacy, diminished sexual frequency, and decreased positive emotions are culprits of honeymoon effects (Baumeister and Bratslavsky, 1999; Jacobs Bao and Lyubomirsky, 2013; Birnbaum et al., 2017; Galak and Redden, 2018).

However, there is some evidence suggesting that marriages may be resilient to the corrosive effects of time. For example, one study showed that in a sample of 1,998 adults examined longitudinally, approximately 40% reported high levels of marital happiness over 20 years (Kamp Dush et al., 2008). Additionally, the happily married group was the most resilient, showing the smallest decreases in life happiness over time. Correspondingly, population studies have shown that approximately 30–40% of individuals in the US married 10 years or more reported high levels of romantic love for their spouse (Acevedo and Aron, 2009; O’Leary et al., 2012). Yet another study demonstrated that couples who idealized each other in the early stages of their relationships were less likely to report steep decreases in love for their partners, measured up to 13 years later (Miller et al., 2006).

To further understand the phenomenon of romantic love maintenance, our overall hypothesis was that romantic love is a developed form of a mammalian drive to find, *and preserve*, preferred mates. Evolution may have selected for diverse strategies in human pair-bonding (some short-term, others long-term) to optimize the chances of offspring survival (Cornwell et al., 2006; Del Giudice et al., 2015; Lim et al., 2015). Our view is consistent with the proposal that love is a complex suite of adaptations that have evolved through sexual reproduction and have, incidentally, turned out to be beneficial beyond mating and bi-parental care of offspring (Buss, 2018). For example, attachment, social bonding, and more generally prosocial behaviors, are thought to have contributed to the advancement of our ancestors through care and cooperation (Baumeister and Leary, 1995).

Thus, we focused on physiological data and studies of non-human mammals as a basis to identify some of the neural and genetic mechanisms involved in sustained romantic love. For example, in monogamous voles the neuropeptides oxytocin (OT) and vasopressin (AVP), and the neurotransmitter dopamine facilitate pair-bonding (e.g., Young et al., 2011). OT and AVP gene polymorphisms, and their receptor-rich brain regions (which are implicated in monogamous pair-bonding), are involved in sexual satisfaction and altruism toward a marriage partner (Acevedo et al., 2019a, b). Moreover, neuroimaging studies by our team, and others, suggest that the mesolimbic reward system is critical for early-stage and long-term romantic love, as well as marital satisfaction (Bartels and Zeki, 2000; Aron et al., 2005; Ortigue et al., 2007; Acevedo et al., 2011, 2012; Xu et al., 2011). Here, we investigated whether these same dopamine-rich reward regions are also involved in the maintenance of romantic love in new marriages.

Several studies have identified genetic polymorphisms associated with pair-bonding. One key polymorphism, *AVPR1a* rs3, has been linked with pair-bonding in voles and humans (Insel et al., 1994; Lim and Young, 2004; Lim et al., 2004; Walum et al., 2008; Jarcho et al., 2011; Acevedo et al., 2019a). In a study of 552 twin pairs and their romantic partners, *AVPR1a* rs3 in men (but not women) was associated with higher levels of partner bonding, fewer relationship problems, greater commitment, and higher levels of relationship quality reported by their romantic partners (Walum et al., 2008). Another study showed that *AVPR1a* rs3 was associated with greater sexual satisfaction and frequency of sexual activity, with corresponding reward system activation in pair-bonded individuals (Acevedo et al., 2019a). More broadly, *AVPR1a* rs3 plays a role in complex social behaviors such as altruism, cognitive empathy, and emotional responsivity to faces (Knafo et al., 2008; Meyer-Lindenberg, 2008; Poulin et al., 2012; Brunnlieb et al., 2016). Thus, we examined the role of *AVPR1a* rs3 in romantic love maintenance.

*OXTR* rs53576, also identified for its role in pair-bonding behaviors (Poulin et al., 2012; Li et al., 2015; Acevedo et al., 2019b), is a single-nucleotide polymorphism (SNP) of the *OXTR* gene that results in individuals having zero, one, or two G-alleles (versus A-alleles). A greater number of G-alleles is associated with more sociality, empathy (Rodrigues et al., 2009; Buffone and Poulin, 2014; Li et al., 2015; Uzefovsky et al., 2015; Gong et al., 2017), and greater altruism toward a partner (Acevedo et al., 2019b). Additionally, the hormone OT is involved in pair-bonding behaviors such as partner hugs, parenting, orgasm, and partner attractiveness ratings (Grewen et al., 2005; Light et al., 2005; Borrow and Cameron, 2011; Striepens et al., 2011; Scheele et al., 2013). Thus, this was another gene polymorphism that we investigated for its role in romantic love maintenance among newlywed pair-bonds.

The dopamine receptor *DRD4*-7R gene variant is associated with novelty-seeking (He et al., 2018, meta-analysis; Munafo et al., 2008, meta-analysis), sexual behaviors such as a desire to have children earlier in life (Eisenberg et al., 2007), desire for a wider variety of sexual behaviors (Halley et al., 2016), higher rates of promiscuous behavior, and infidelity (Garcia et al., 2010). The *DRD4-*7R genetic polymorphism results in reduced binding for dopamine (Asghari et al., 1995), and thus some have speculated that individuals with this genetic variant generally feel less stimulated and crave novelty (He et al., 2018). Although our examination of *DRD4-*7R was exploratory, prior research studies suggest that this genetic variant is implicated in short-term pair-bonding strategies (Minkov and Bond, 2015) which are useful for reproduction, but a potential obstacle for relationship maintenance. Dopamine is also involved in pair-bonding in voles (Young et al., 2011) and dopamine-rich brain sites have been shown in association with both early-stage and long-term love (see Acevedo, 2015, review). Thus, we examined DRD4 as an indicator of the dopamine system’s involvement in romantic love, which has been inferred in research on romantic love, but only tested in a few studies (i.e., Takahashi et al., 2015).

Another gene that affects dopamine transmission in the brain is *COMT*. *COMT* codes for catechol-*O*-methyltranferase (COMT), an enzyme which degrades catecholamines, including dopamine, as they are released in the synapse (Männistö and Kaakkola, 1999). *COMT* rs4680, one allelic variant of *COMT*, results in increased COMT activity and thus lower dopamine levels. Individuals with the *COMT* rs4680 A- (versus G) allele variant have decreased COMT activity resulting in higher dopamine levels (Chen et al., 2004). Thus, they show greater reward-seeking behavior and reward responsiveness, and higher subjective ratings of pleasure in response to positive events, compared to those with more G-alleles (Wichers et al., 2007; Lancaster et al., 2012). One study with 120 participants found that individuals with more COMT A-alleles scored higher on the “Temporal Experience of Pleasure Scale” (Gard et al., 2006), which measures trait anticipatory and consummatory positive affect (Katz et al., 2015). These effects were mediated by activation of the prefrontal cortex in the post-reward phase, suggesting links between *COMT* A-alleles and self-reported consummatory positive affect. Furthermore, in a meta-analysis of 51 studies, a greater number of *COMT* rs4680 A-alleles were associated with obsessive compulsive disorder in males (Taylor, 2016). Obsessive compulsive disorder is correlated with dopaminergic activation (Goodman et al., 1990; Denys et al., 2004), and obsessive thinking is characteristic of romantic infatuation which includes intrusive, uncontrollable thoughts about the partner (Tennov, 1999). Thus, we examined *COMT* rs4680 as a marker for sensitivity to dopaminergic action and potentially romantic love.

Building on human and animal studies examining the biological underpinnings of pair-bonding, this study investigated the neural and genetic correlates underlying romantic love maintenance over the first year of newlywed marriages. We measured self-report (Eros scale) and neural (functional MRI) correlates of romantic love among first-time newlyweds, observed around the wedding date (T1) and 1-year after (T2), implementing a scanning protocol used in prior studies examining romantic love (Aron et al., 2005; Acevedo et al., 2011). The fMRI task measured participants’ neural activity in response to viewing facial images of their partner versus a familiar, neutral acquaintance. We defined romantic love maintenance as stability in Eros scores (i.e., small change) between T1 and T2. Each individuals Eros difference score (T2-T1) was correlated with brain activations at T1 to determine what brain systems might be predictive of romantic love maintenance, and at T2 to determine what brain systems might be involved in the maintenance of romantic love. We focused our results on the brain activations that were shown at both T1 and T2, but also made available T1 and T2 results in Supplementary Tables.

Also, for the first time in human romantic love studies, we analyzed interactions of romantic love maintenance with genetic polymorphisms (*AVPR1a* rs3, *OXTR* rs53576, *DRD4-*7R, and *COMT* rs4680) implicated in monogamous pair-bonding. We predicted that neural, hormonal, and genetic correlates of pair-bonding found in other mammals, and the brainstem reward/drive system identified in human love studies, would be involved in the maintenance of romantic love over first-year marriages. Beyond advancing the science of pair-bonding, such findings might also benefit couples and therapists through a deeper understanding of the processes that sustain romantic love.

## Materials and Methods

### Participants

Participants provided informed consent in accordance with the IRB procedures of the University of California, Santa Barbara (UCSB) and Albert Einstein College of Medicine. Subjects were recruited via advertisements, flyers, listservs, and word of mouth. Before undergoing scanning, all participants were interviewed to assess eligibility criteria such as first-time marriage for both partners, no children for either partner, relatively good health, and fMRI contraindications (right-handedness, good health, no metal in or on the body, no claustrophobia, no pregnancy, and no history of head trauma). The eligibility criteria were selected to reduce variability of the sample since this was the first study to examine the physiology underlying changes in romantic love in newlywed marriages. We selected individuals in first-time marriages with no children to mirror animal studies where monogamous mammals solidify pair-bonds prior to producing offspring. All procedures were described at the time of the interview.

The resulting sample was composed of 19 (11 women and 8 men) healthy, right-handed individuals, ages 21 to 32 (27.21 ± 3.29 years), in established relationships (4.11 ± 3.09 years), without children, and living with their partner about 2 years (1.9 ± 1.6 years). At baseline (T1), some participants were recently married (10 married, 1.9 ± 1.5 months), while others were soon to be married (2.6 ± 1.7 months until the wedding). The sample of participants were mostly college-educated: 11 participants had college degrees and 6 had a master’s degree or higher, while only 2 had a high-school level education. The mean annual household income of the sample was $62,000 (±$28,000, range $16,000 to $110,000).

Thirteen (seven females and six males) of the 19 participants returned for a second scan (T2), approximately 11.3 months (SD ± 1.3, range 9.0–13.5) after the initial scan (T1). Herein, we report findings that were shown at both measurements (T1 and T2) among the group of 13 participants that were scanned twice.

### Procedure

Once eligibility was confirmed, participants provided the experimental stimuli: face images of their partner and a highly familiar neutral acquaintance (HFN). The HFN served as a control for facial familiarity and was matched to the partner by age, sex, and length of time known. The partner-HFN face viewing task has been used in prior fMRI studies of romantic love (e.g., Aron et al., 2005; Xu et al., 2011) and was originally developed in a study showing that images of partner faces, compared to other types of stimuli (i.e., songs and scents), elicited the most intense love feelings among individuals in love (Mashek et al., 2000). All photos were digitized according to standard procedures where only the face was presented, and they were shown with Presentation software (Psychological Software Tools, Inc., Pittsburgh, PA, United States) during the scan.

For the fMRI task, participants viewed alternating face images of the partner and the HFN (shown individually) interspersed with a countback task (displayed individually, for 20 s each). For the countback task (used to reduce carry-over effects of viewing the facial images), subjects were asked to mentally count backwards in increments of seven, starting with a random four-digit number displayed on the screen. The entire session lasted for 12 min, and stimuli (images and the countback task) were displayed for 20 s each. At the start of the session, participants were instructed to recall non-sexual events with the person whose face image would be displayed on the screen. After the scan, participants provided affective ratings while still in the scanner to verify that the evoked emotion corresponded to the target image (see Acevedo et al., 2014 for results of the affective ratings). Identical procedures, questionnaires, and photos were used at T1 and T2.

#### Questionnaires

Participants completed a battery of questionnaires, including the Eros measure of romantic love from the Love Attitudes Scale (LAS; Hendrick and Hendrick, 1986), the widely used Relationship Assessment Scale (RAS, Hendrick, 1988) for relationship satisfaction, and two items assessing sexual satisfaction and frequency. All measures used a 1–7 item Likert rating scale. Descriptive statistics and correlations are shown in Tables 1, 2.

**TABLE 1:** Relationship self-report mean (*M*) and standard deviations (*SD*).

	**T1**		**T2**			
	***M***	***SD***	***M***	***SD***	***T***	***p***
Romantic love	6.33	0.32	6.17	0.87	0.55	0.59
Relationship satisfaction	6.33	0.59	6.35	0.57	0.13	0.90
Sexual satisfaction	5.85	0.90	5.23	1.54	1.28	0.19
Sexual frequency/week	2.95	1.87	1.83	1.25	4.39	<0.01

**TABLE 2:** Correlations among self-report relationship measures and gene polymorphisms in newlyweds.

**Variables**	**1**	**2**	**3**	**4**	**5**	**6**	**7**	**8**	**9**	**10**	**11**	**12**
(1) Romantic love T1	–											
(2) Relationship satisfaction T1	0.38	–										
(3) Sexual satisfaction T1	0.19	–0.27	–									
(4) Sexual frequency T1	0.55*	0.30	0.24	–								
(5) Romantic love T2	0.21	0.65*	0.09	0.16	–							
(6) Relationship satisfaction T2	0.05	0.83**	–0.27	0.12	0.73**	–						
(7) Sexual satisfaction T2	–0.12	0.30	0.21	0.11	0.74*	0.45	–					
(8) Sexual frequency T2	0.50*	0.33	0.41	0.90**	0.21	0.16	0.28	–				
(9) *AVPR1a* rs3	0.26	0.58*	–0.11	–0.12	0.46*	0.38	0.12	0.04	–			
(10) *OXTR* rs53576	0.29	0.08	0.25	0.12	0.31	0.23	–0.01	0.14	0.07	–		
(11) *DRD4*-7R	−0.81**	–0.37	–0.06	–0.41	–0.27	0.04	0.00	–0.35	–0.33	–0.08	–	
(12) *COMT* rs4680	−0.50^†^	–0.35	0.33	0.02	0.10	–0.05	0.43	0.14	–0.34	0.38	0.45^†^	–

*p < 0.05, **p < 0.01, ^†^marginally significant *p* < 0.10.

The LAS measures six different types of love attitudes toward one’s romantic partner: romantic love (Eros), obsessive love (Mania), game-playing love (Ludus), friendship-love (Storge), practical love (Pragma), and altruistic love (Agape). The LAS has been shown to reliably measure these six different love factors (Cronbach’s α = 0.39 to 0.87; Shahrazad et al., 2012). Here, we report results for the Eros scale since our focus was on romantic love without infatuation/obsession (see Acevedo and Aron, 2009). Sample Eros scale items include, “My partner and I have the right physical chemistry between us,” “My partner and I really understand each other,” and “I feel that my partner and I were meant for each other” (Cronbach’s α = 0.40, 0.72 at T1 and T2, respectively).

The RAS is a seven-item unifactorial measure of relationship satisfaction with items such as, “How well does your partner meet your needs?” and “To what extent has your relationship met your original expectations?” (Cronbach’s α = 0.68, 0.89 at T1 and T2, respectively).

Sexual satisfaction was assessed with one item: “How happy are you with your sex life with your partner?” Sexual frequency used one item: “How frequently do you and your partner engage in sexual activity?”

#### Gene Sampling and Analysis

Subjects provided saliva samples for DNA extraction via Oragene test tubes. Detection of the number of repeats for *AVPR1a* rs3 and the *DRD4*-7R 48 base-pair repeat sequence was performed using fragment analysis, in which repeat sequences are specified using sequence-specific primers and amplified for detection using polymerase chain reaction (PCR). For the present study, PCR was performed on 50 ng of DNA in buffer [100 mM Tris-HCl (pH 8.0), 500 mM KCl, 1.5 mM MgCl2, 0.2 mM dNTP, 0.2 μM of each primer, and 1 unit of TaqPolymerase (Applied Biosystems)]. Cycling conditions were the following: initial denaturation at 95°C for 2 min followed by 30 cycles of denaturation at 94°C for 30 s, annealing at 55°C for 30 s, and extension at 72°C for 45 s, with a 15 min final extension at 72°C. Microsatellite fragment analyses of RS3 and the *DRD4*-7R polymorphism (i.e., identifying the number of repeats for each sequence) were performed using the ABI 3730 DNA analyzer and Genemapper 3.5 software (Applied Biosystems). For *AVPR1a* rs3, the number of repeat sequences was split at the median (*M* = 335.86 ± 2.87, range = 330.93 – 341.30) to designate each allele as “long” versus “short.” The number of long alleles (0, 1, or 2) was used as a continuous variable in our analyses.

Genotyping of the *OXTR* rs53576 and *COMT* rs4680 SNPs was conducted using the MassARRAY Compact system on a panel of custom SNP assays designed using RealSNP and MassARRAY Assay Designer (Sequenom Inc.). The protocol involved PCR amplification of 10 ng DNA using SNP-specific primers followed by a base extension reaction using iPLEX gold chemistry (Sequenom Inc.). The final base extension products were treated and spotted on a 384-pad SpectroCHIP using a ChipSpotter LT nanodispenser (Samsung). A MassARRAY Analyzer Compact MALDI-TOF-MS was used for the data acquisition process from the SpectroCHIP. The resulting polymorphisms were called using MassARRAY Typer Analyzer v4.0 (Sequenom, Inc.), and the number of G- or A-alleles was used as a continuous variable in our analyses.

### Imaging Data Acquisition and Analysis

A 3.0 T Siemens Trio with a 12-channel phased-array head coil was used for the acquisition of blood oxygenation level dependent (BOLD) responses. A single-shot echo planar imaging sequence sensitive to BOLD contrast was used to acquire 37 slices per repetition time (TR = 2000 ms, 3 mm thickness, 0.5 mm gap), with an echo time (TE) of 30 ms, flip angle of 90 degrees, field of view (FOV) of 192 mm, and 64 × 64 acquisition matrix. Prior to the acquisition of BOLD responses, a high-resolution T1-weighted sagittal sequence image of the whole brain was obtained (TR = 15.0 ms; TE = 4.2 ms; flip angle = 9 degrees, 3D acquisition, FOV = 256 mm; slice thickness = 0.89 mm, acquisition matrix = 256 × 256).

#### Imaging Data Processing

Functional images were subjected to standard preprocessing procedures using SPM5 (Wellcome Department of Cognitive Neurology). First, functional EPI volumes were realigned to the first volume, smoothed with a Gaussian kernel of 6 mm, and then normalized to the T1.nii image template. No participant showed movement greater than 3 mm (whole voxel). After pre-processing, the partner-versus-HFN contrasts were created separately for the T1 and T2 group results.

#### Multiple Regression Data Analysis

Multiple regression analyses were carried out to estimate group brain activity associated with (a) romantic love difference scores (T2 minus T1) and (b) interactions between romantic love difference scores (T2-T1) with *AVPR1a* rs3, *OXTR* rs53576, *COMT* rs4680 and *DRD4*-7R, examining each gene separately. The effects of *AVPR1a*, *OXTR*, *COMT*, and *DRD4* were tested in separate models. Thus, results are presented for each separate regression. There were no significant differences in sex, age, or relationship length therefore, analyses were conducted without controlling for these variables.

#### Regions of Interest (ROIs) and Whole-Brain Analyses

Regions of interests for the activations were based on previous studies of romantic love (noted in the table legends). We adopted a false discovery rate (FDR) for multiple comparisons correction (Genovese et al., 2002) at *p* < 0.05. ROIs occupied a 3–10-mm radius with a 3-voxel minimum, depending on the size of the brain area. For exploratory purposes, we conducted whole-brain analyses at *p* < 0.001 (uncorrected for multiple comparisons), minimum spatial extent of >5 contiguous voxels. All regions were confirmed using the human brain atlas by Mai et al. (2016). Tables 3–5 report significant effects replicated at T1 and T2 to minimize the risk of false positive findings due to our small sample size. Other results are reported in the Supplementary Tables.

**TABLE 3:** Regional brain activations correlated with romantic love maintenance among newlyweds.

	**Left**					**Right**				
**Brain Region**	***x***	***y***	***z***	***T***	***p***	***x***	***y***	***z***	***T***	***p***
Brain responses replicated at Times 1 and 2										
*ROI Activations*										
SN, lateral^1^						15	−15	−12	3.97	0.001
Paracentral lobule^2^	−6	−24	57	3.45	0.01					
*Whole-brain Deactivations*										
Inferior frontal gyrus						54	21	3	4.13	<0.001

*All results are for regions showing greater activation in associated with change in Eros scores (T2-T1) over the first year of marriage in newlyweds. Superscripts denote references for ROIs: ^1^Acevedo et al. (2011); ^2^Wise et al. (2016).*

**TABLE 4:** Regional brain activations showing interactions with *AVPR1a* rs3 (long alleles) and romantic love maintenance among newlyweds.

	**Left**					**Right**				
**Brain Region**	***x***	***y***	***Z***	***T***	***p***	***x***	***y***	***z***	***T***	***p***
Brain responses replicated at Times 1 and 2										
*ROI Activations*										
VTA, posterior^1^						6	−21	−21	2.58	0.02
Periaqueductal gray^1^						3	−33	−21	2.87	0.02
Posterior hippocampus^1^						39	−27	−9	3.87	0.01
Occipital cortex, area 17/18^1^						15	−90	3	2.49	0.02
*Whole-brain Activations*										
Superior temporal gyrus/Angular gyrus						45	−78	24	4.41	<0.001

*All results are for regions showing activation in association with *AVPR1a* rs3 long versus short alleles and change in Eros scores (T2-T1) over the first year of marriage in newlyweds. Superscripts denote references for ROIs: ^1^Acevedo et al. (2011).*

**TABLE 5:** Regional brain activations showing interactions with *OXTR* rs53576 (G alleles) and romantic love maintenance among newlyweds.

	**Left**					**Right**				
**Brain Region**	***X***	***Y***	***Z***	***T***	***p***	***x***	***y***	***z***	***T***	***p***
Brain responses replicated at Times 1 and 2										
*ROI Activations*										
VTA/SN^1^	−3	−15	−21	4.43	0.01					
Septum/fornix region^1,2^	0	0	23	3.83	0.01	3	0	24	3.64	0.02

*All results are for regions showing activation in association with *OXTR* rs53576 (G versus A alleles) and change in Eros scores (T2-T1) over the first year of marriage in newlyweds. Superscripts denote references for ROIs: ^1^Acevedo et al. (2011); ^2^Aron et al. (2005).*

## Results

### Descriptive Statistics

Descriptive statistics are reported in Table 1. The mean change in Eros scores from T1 to T2 (*M* = −0.13 ± 0.89, range = −3.00 to +0.50) was not statistically significant. Specifically, 75% of the sample showed increases of less than a point, 25% showed no change, and 25% showed decreases of less than a point in Eros scores. Thus, the majority of the sample reported romantic love maintenance. Only one participant showed a steep decrease (−3.00 points) in romantic love over the first year of marriage. Thus, we examined the data without the outlier. However, the brain imaging correlations did not change significantly, including the *OXTR, AVPR1a*, *DRD4*, and *COMT* interactions with romantic love maintenance (Eros T2-T1). Additionally, activation of the VTA in response to images of the partner remained positive, but in some cases became non-significant, when the outlier was excluded. Thus, we proceeded with analyses including the outlier because variable values make these results more generalizable to the population. That is, it is expected that some couples will experience steep decreases in romantic love in the early stages of marriage as shown by research reporting “honeymoon effects” (e.g., Huston et al., 2001).

### Correlations Among Variables

Correlations among self-report measures are reported in Table 2. At each time point, romantic love was significantly correlated with frequency of sexual activity (T1: *r* = 0.55, *p* < 0.05; T2: *r* = 0.50, *p* < 0.05). Romantic love was also strongly correlated with relationship satisfaction: at T1 relationship satisfaction predicted romantic love at T2 (*r* = 0.65, *p* < 0.05), and at T2 relationship satisfaction was correlated with romantic love at T2 (*r* = 0.73, *p* < 0.01). Gene correlations showed that *AVPR* rs3 (long alleles) was significantly correlated with relationship satisfaction at T1 (*r* = 0.58, *p* < 0.05) and with romantic love at T2 (*r* = 0.46, *p* < 0.05). Also, both dopamine polymorphisms, *DRD4*-7R (*r* = −0.81, *p* < 0.01) and *COMT* rs4680 (*r* = −0.50, *p* < 0.10), were negatively correlated with romantic love scores at T1.

#### Gene Polymorphism Distributions

Gene polymorphism distributions for the sample were as follows: *AVPR1a* rs3 (short = 4, short/long = 6; long = 3), *OXTR* rs53576 (AA = 1, AG = 6, GG = 6), *COMT* rs4680 (AA = 2, AG = 6, GG = 5), and *DRD4*-7R (2 = 2 repeats, 2 = 3 repeats, 7 = 4 repeats, 2 = 7 repeats).

### Neuroimaging Results

#### Neural Correlates of Romantic Love Maintenance

**FIGURE 1:**
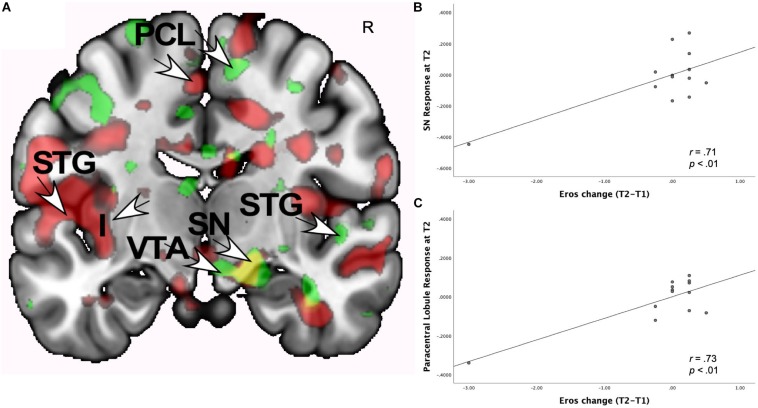
**(A)** Regional brain activations showing positive correlations with change in romantic love scores (T2-T1: love maintenance) over the first year of marriage among newlyweds in response to viewing face images of the partner versus a highly familiar, neutral acquaintance. Yellow: T1 *and* T2 brain responses in the right SN (arrow). Red: T2 brain responses in the R anterior VTA (arrow) and R STG (arrow). Green: T1 brain responses in the L STG (arrow), and L PCL (arrow). **(B)** Scatterplot shows the correlation between change in romantic love (Eros) scores (T2-T1) and R SN activation at T2. **(C)** Scatterplot shows the correlation between change in romantic love scores (T2-T1) and L PCL activation at T2.

As Table 3 shows, at both T1 and T2 neural responses to the partner (versus HFN) images showed significant correlations with romantic love maintenance (Eros T2-T1 scores) in the right SN and the left paracentral lobule (PCL) (see Figure 1A). Scatterplots show correlations between Eros scores and activity in the right SN and PCL at T2 (Figures 1B,C). Significant deactivation at both T1 and T2 was observed in the inferior frontal gyrus (IFG).

Some activations occurred only at T1 or T2, but not both. At T1-only, partner (versus HFN) activations predictive of romantic love maintenance (T2-T1 Eros scores) were observed in the raphe, pons, medial prefrontal cortex, and paracentral lobule (ROIs), as well as the right perirhinal/fusiform, superior frontal gyrus (SFG), superior temporal gyrus (STG) and the left precuneus (whole-brain). At T2-only, partner (versus HFN) activations were significantly correlated with romantic love maintenance (T2-T1 scores) in the right amygdala/globus pallidus (GP) and the left mid-insula (ROIs); and the bilateral occipital cortex, supplementary motor area, precentral gyrus, left and right SFG, and parietal area (whole-brain). At T1-only, a number of deactivations were observed in the right anterior insula (AI), occipital cortex, middle frontal gyrus (MFG), and the left collateral sulcus. At T2-only, deactivations were prominent in the right SFG and the left angular gyrus (AG) (see Supplementary Table 1).

#### Neural Interactions of Romantic Love × *AVPR1a* rs3

**FIGURE 2:**
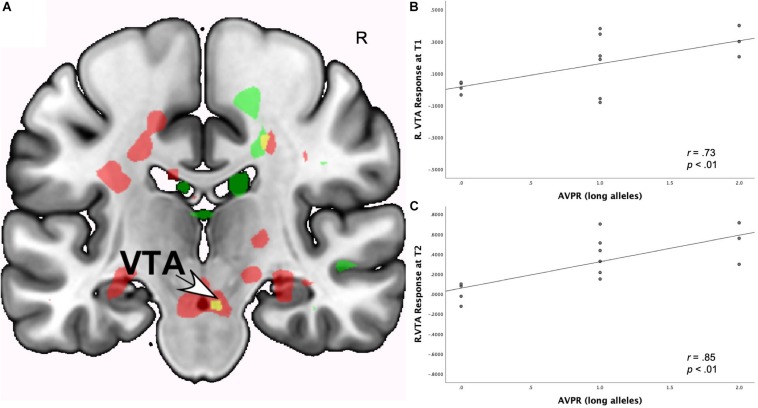
**(A)** Regional brain response interactions with *AVPR1a* rs3 (long versus short alleles) and change in romantic love scores (T2-T1: love maintenance) among newlyweds in response to viewing facial images of the partner versus a highly familiar, neutral acquaintance. Yellow: T1 *and* T2 brain responses in the R posterior VTA (arrow). Red: T2 brain responses bilaterally in the posterior VTA. **(B)** Scatterplot shows the correlations between *AVPR1a* rs3 (long versus short alleles) and the R VTA response at T1. **(C)** Scatterplot shows the correlations between *AVPR1a* rs3 (long versus short alleles) and the R VTA response at T2.

As shown in Table 4, at both T1 and T2, the interaction of romantic love maintenance with *AVPR1a* rs3 (long versus short alleles) showed significant effects in the right posterior VTA (Figures 2A–C), the PAG, posterior hippocampus, occipital cortex (ROIs), and the STG (whole-brain). Scatterplots show the correlations between *AVPR1a* rs*3* and the right VTA response at T1 and T2 (Figures 2B,C).

As shown in Supplementary Table 2, T1-only partner (versus HFN) activations were predictive of romantic love maintenance as a function of *AVPR1a* rs3 (long versus short alleles) in the right caudate tail, pons, septum fornix, and amygdala/GP (ROIs). At T2-only, interactions with *AVPR1a* rs3 were shown in the left VTA, caudate head, bilateral raphe, hippocampus/caudate tail, posterior hippocampus, left anterior cingulate cortex (ACC), occipital cortex (ROIs), and the right lateral geniculate (whole-brain).

#### Neural Interactions of Romantic Love x *OXTR* rs53576

**FIGURE 3:**
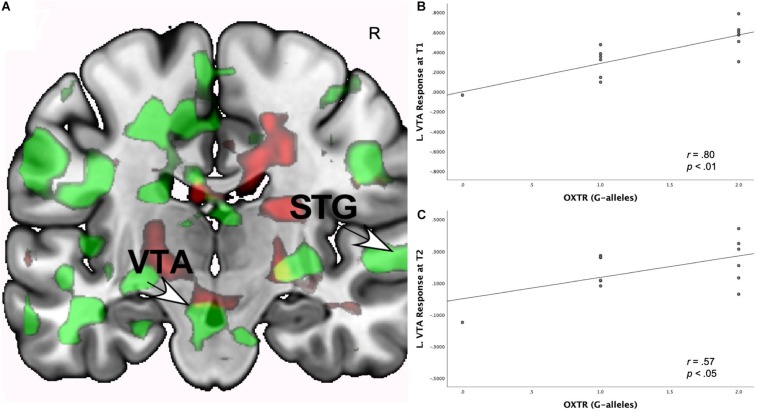
**(A)** Regional brain response interactions with *OXTR* rs53576 (G versus A-alleles) and change in romantic love scores (T2-T1: love maintenance) among newlyweds in response to viewing facial images of the partner versus a highly familiar, neutral acquaintance. Yellow: T1 *and* T2 responses in a small part of the left VTA (arrow). Green: T1 brain response bilaterally in the posterior VTA/SN. Red: T2 brain response bilaterally in the posterior VTA/SN. **(B)** Scatterplot shows the correlations between *OXTR* rs53576 (G versus A-alleles) and the L VTA response at T1. **(C)** Scatterplot shows the correlations between *OXTR* rs53576 (G versus A-alleles) and the L VTA response at T2.

As shown in Table 5, at both T1 and T2, the interaction of romantic love maintenance with *OXTR* rs53576 (G versus A-alleles) showed significant effects in the left VTA/SN and bilateral septum/fornix (Figure 3A).^1^ Scatterplots show the correlations between *OXTR* rs53576 with left VTA responses at T1 and T2 (Figures 3A–C).

As shown in Supplementary Table 3, T1-only partner (versus HFN) activations predicted romantic love maintenance (T2-T1) as a function of *OXTR* rs53576 (G versus A-alleles) in the right PAG, basolateral amygdala, left central amygdala, hippocampus (ROIs), and the bilateral occipital/lingual gyrus (whole-brain). At T2-only, activations as a function of *OXTR* rs53576 (G versus A-alleles) were observed in the left posterior VP, caudate, right central amygdala (ROIs), and the right intraparietal sulcus, IFG, MFG, STG, and left dorsolateral PFC (whole-brain). Deactivations were evident at T1 in the bilateral SFG and left MFG. At T2-only, deactivations were observed in the left caudate, AG, somatosensory cortex, and bilaterally in the lateral geniculate and premotor cortex.

#### Neural Interactions of Romantic Love x *DRD4*-7R

**FIGURE 4:**
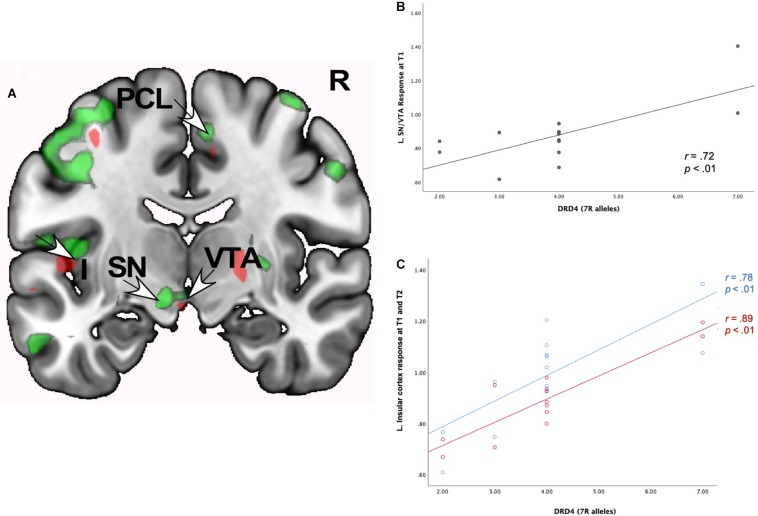
**(A)** Regional brain response interactions with *DRD4* 7R alleles and change in romantic love scores (T2-T1: love maintenance) among newlyweds in response to viewing facial images of the partner versus a highly familiar, neutral acquaintance. Red: T2 brain responses in the anterior VTA (bottom right arrow) and insular cortex (leftmost arrow). Green: T1 brain response in the anterior VTA/SN (left bottom arrow), insular cortex (leftmost arrow), and R PCL (top arrow). **(B)** Scatterplot shows the correlations between *DRD4* 7R alleles and the L VTA response at T1. **(C)** Scatterplot shows the correlations between *DRD4*-7R alleles and the L insular cortex response at T1 and T2.

As Table 6 shows, at both T1 and T2, romantic love maintenance was positively correlated with *DRD4* (greater number of 7R alleles) and activity in the left VTA/SN and posterior insular cortex (Figures 4A–C). Scatterplots show the correlations between *DRD4-*7R with activation in the left SN/VTA at T1, and the insular cortex at T1 and T2 (Figures 4A–C).

**TABLE 6:** Regional brain activations showing interactions with *DRD4* 7R alleles and romantic love maintenance among newlyweds.

	**Left**					**Right**				
**Brain Region**	**x**	**y**	**Z**	**T**	***p***	**x**	**y**	**z**	**T**	***p***
Brain responses replicated at Times 1 and 2										
*ROI Activations*										
VTA/SN^1^	−9	−12	−9	3.26	0.01					
Insular cortex^2,3^	−45	−12	9	3.85	0.01					
*Whole-brain Deactivations*										
Temporal gyrus, anterior	−39	9	−24	6.29	<0.001					

*All results are for regions showing activation in association with *DRD4* 7R alleles and change in Eros scores (T2-T1) over the first year of marriage in newlyweds. Superscripts denote references for ROIs: ^1^Acevedo et al. (2011); ^2^Aron et al. (2005); ^3^Xu et al. (2011).*

At T1-only, partner (versus HFN) activations were predictive of romantic love maintenance as a function of *DRD4*-7R in the bilateral medial PFC, right PCL (ROIs), and the right dorsolateral PFC (DLPFC), entorhinal cortex, left SI, supramarginal gyrus, and lateral PFC (whole-brain). At T2-only, activations as a function of *DRD4*-7R were observed in the left somatosensory cortex and the DLPFC (whole-brain). Deactivation in the left temporal gyrus was evident at both T1 and T2, and at T2 deactivations were observed in the bilateral hippocampus and the right temporal gyrus (see Supplementary Table 4).

#### Neural Interactions of Romantic Love × *COMT* rs4680

**FIGURE 5:**
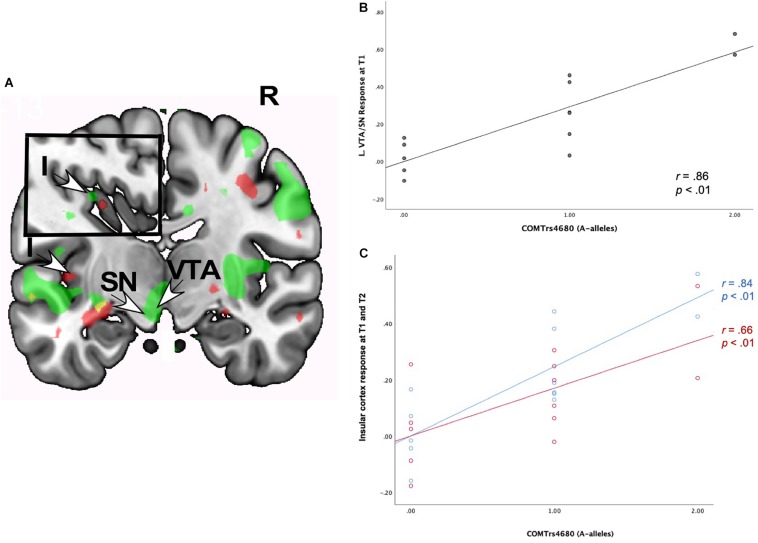
**(A)** Regional brain response interactions with *COMT* rs4680 (A versus G alleles) and change in romantic love scores (T2-T1: love maintenance) among newlyweds in response to viewing face images of the partner versus a highly familiar, neutral acquaintance. Inset shows a sagittal view of the insular cortex. Green: T1 brain response in the VTA/SN (bottom arrows) and insular cortex (inset). Red: T2 responses in the insular cortex (left arrow and inset). **(B)** Scatterplot shows the correlations between *COMT* rs4680 (A versus G alleles) and L VTA/SN response at T1. **(C)** Scatterplot shows the correlations between *COMT* rs4680 (A versus G alleles) and L insular cortex response at T1 and T2. R, right. L, Left. I, insular cortex; SN, substantia nigra; VTA, ventral tegmental area. Other colored regions did not meet the statistical requirements for whole-brain analysis or were not an ROI. Yellow, significant correlations at T1 and T2. Green, significant correlations at T1. Red, significant correlations at T2.

As shown in Table 7, romantic love maintenance, at both T1 and T2, was positively correlated with *COMT* rs4680 (greater number of A-alleles) and response to partner (versus HFN) images in the left SN/VTA and posterior insular cortex (Figures 5A–C). Scatterplots show the correlations between *COMT* rs4680 with the left SN/VTA response at T1, and with insular cortex response at T1 and T2 (Figures 5A–C).

**TABLE 7:** Regional brain activations showing interactions with *COMT* rs4680 (A-alleles) and romantic love maintenance among newlyweds.

	**Left**					**Right**				
**Brain Region**	**x**	**y**	**Z**	**T**	***p***	**x**	**y**	**z**	**T**	***p***
Brain responses replicated at Times 1 and 2										
*ROI Activations*										
SN/VTA^1^	−6	−12	−12	5.10	0.01					
Insular cortex^2,3^	−42	−18	6	4.56	0.01					

*All results are for regions showing activation in associated with *COMT* rs4680 (A versus G-alleles) and change in Eros scores (T2-T1) over the first year of marriage in newlyweds. Superscripts denote references for ROIs: ^1^Acevedo et al. (2011); ^2^Aron et al. (2005); ^3^Xu et al. (2011).*

Supplementary Table 5 shows T1-only partner (versus HFN) activations predictive of romantic love maintenance (T2-T1) as a function of *COMT* rs4680 in the bilateral medial PFC, the right primary sensory cortex, and the left secondary somatosensory cortex (whole-brain). At T2-only, activations as a function of *COMT* rs4680 were shown in the left PCL (ROI); and in the right VLPFC, DLPFC, and posterior cingulate cortex (whole-brain). Additionally, deactivations were observed in the hippocampus at T1, while at T2 deactivations were evident in the SN, caudate tail, and dorsal midbrain.

## Discussion

We investigated the neural and genetic (*AVPR1a* rs3, *OXTR* rs53576, *DRD4*-7R and *COMT* rs4680) correlates of romantic love maintenance among first-time newlyweds. Marriage is a pivotal life event that marks the establishment of the family unit, with implications for reproduction, bi-parental care of offspring, long-term companionship, and well-being (Fletcher et al., 2015). Using fMRI, we scanned newlyweds around the time of the wedding (T1), and a subset returned for a second scan about 1 year later (T2). Consistent with research on the neural correlates of long-term romantic love (Acevedo et al., 2011), at both time points, newlyweds showed activation in the dopamine-rich substantia nigra (SN) in association with romantic love maintenance. They also showed dopamine-rich, VTA-related genetic expression in association with *AVPR1a* rs3 (right side), *OXTR* rs53576 (left side), *DRD4*-7R (left side), and *COMT* rs4680 (left side) with romantic love maintenance at both time points. The VTA effects were stronger for *AVPR1a* rs3 long-alleles and *OXTR* rs53576 G-alleles. These genes are associated with complex social behaviors including pair-bonding (Walum et al., 2008; Li et al., 2015; Gong et al., 2017). Interestingly, the VTA-related *AVPR1a* and *OXTR* effects were observed in a different area, more posterior to those shown for the simple correlation with romantic love maintenance and the dopamine (DRD4 and COMT) genetic interaction effects. This suggests functional segregation of the VTA/SN with different density receptors for OT and AVP compared to dopamine.

The VTA/SN reward regions are involved in coordinating primary behaviors needed for survival and reproduction, such as mating and feeding. They are also involved in secondary reward-processing including responses to monetary gains and addictive substances (Risinger et al., 2005; Fields et al., 2007; D’Ardenne et al., 2008). Largely mediated by dopamine neurons, VTA/SN activity affects reward-seeking, motivation, “wanting,” and the drive to “work” for rewards (Berridge and Robinson, 2003). An extensive body of research has shown that dopamine neurons modulate approach-related behaviors, response to novel stimuli, and euphoric experiences (Berridge and Robinson, 2003; Childress et al., 2008; Georgiadis et al., 2010; Schultz, 2010; Krebs et al., 2011; Ikemoto et al., 2015; Noori et al., 2016). Consistent with previous work (for reviews, see Ortigue et al., 2010; Acevedo, 2015), and expanding on it, these findings highlight how the brain’s reward system mediates behaviors that are critical for romantic love and its maintenance over time, such as proximity-seeking, positive affect, continued desire, and engaging in relationship-promoting behaviors (such as doing things that make a partner happy).

The present findings also provide the first direct evidence that dopamine-related gene expression in the VTA/SN is involved in the maintenance of romantic love in humans. Previous fMRI studies of romantic love assumed that the VTA response reflected dopamine activation (Aron et al., 2005). Although one study showed dopamine release in the prefrontal cortex while viewing the face of a new romantic partner (Takahashi et al., 2015), here we used genetic markers to confirm direct involvement of dopamine in the midbrain/VTA. Interestingly, our results are consistent with a recent study which showed that dopamine-related gene expression in the VTA of male zebra finches was associated with pair-bonding behaviors (nesting and courtship) of their female pair (Alger et al., 2020). Also, individual differences in social interactions in long-term zebra finch pairs were associated with the expression of several dopamine-related genes in the VTA. Collectively, these findings highlight the important function of the midbrain VTA region and dopamine for pair-bonding and romantic love.

Additionally, our findings are consistent with the dopamine hypothesis of romantic love (Fisher et al., 2006) and theories suggesting that romantic love is a motivational drive akin to a “natural” addiction (Frascella et al., 2010; Fischer et al., 2016), but also different from drug addiction (Wang et al., 2020). Thus, in addition to advancing knowledge on the biological factors underlying romantic love maintenance, these findings may also be applied to other fields such as the study of the maintenance of “natural” reward/addictions/cravings.

### Sex and Romantic Love Maintenance

Other notable findings shown in the present group of newlyweds in association with romantic love maintenance emerged in regions important for sex and sensory processes (the PCL and sensory cortex). Interestingly, the PCL is the genital sensorimotor region activated in women during orgasm and clitoral stimulation (Wise et al., 2016, 2017). Activation of the PCL in the present study is interesting because the primary sensory cortex (SI) usually requires direct tactile stimulation to activate it. There was no stimulation of the genitals in this study, and participants were instructed *not* to think about sexual memories. The traditional textbook understanding of SI function does not include memory, emotion, or person representation, only sensory processing features like pressure. However, studies in recent years suggest that SI may contain memory capacity and a genetically controlled mechanism for cortical memory (Bancroft et al., 2014; Kragel and LaBar, 2016; Muckli and Petro, 2017; Galvez-Pol et al., 2018; Kim et al., 2018). Finally, there is substantial evidence that a memory code for *persons and traits* is active in the ventral medial prefrontal cortex, while other cortical areas are also involved in the mental representation of a person (Heleven and Van Overwalle, 2016; Thornton and Mitchell, 2018). We speculate that looking at the face of the marriage partner and thinking romantic thoughts might activate the mental representation of that person, as faces have in other studies (e.g., Thornton and Mitchell, 2018). Additionally, it’s likely that that engaging in sexual acts with the same partner over time would activate genital sensory cortex memory storage mechanisms that importantly become part of the mental representation of the partner.

Although many therapists have suggested an important role for sexual activity in maintaining a marital relationship, this is the first time a cortical brain region associated with direct sexual stimulation has been correlated with self-reports of romantic love in marriages while simply thinking (and viewing face images) of a spouse. Further support for the importance of sex emerged from the robust correlations between romantic love scores and sexual frequency and satisfaction ratings at both time points (see Table 2).

### *AVPR1a* and Romantic Love Maintenance

Interestingly, the present sample of newlyweds showed significant interactions with *AVPR1a* rs3 and romantic love maintenance, at both time points, in the right VTA (Figures 2A–C), the PAG, posterior hippocampus, occipital cortex and the superior temporal gyrus (STG)— regions important for reward, attachment, memory, and visual and sensory processing (Nagy et al., 2012; for a meta-analysis, see Phan et al., 2002; Schultz et al., 2003; Wager et al., 2003). Most of these regions have appeared in the context of long-term romantic love and maternal love (e.g., Acevedo et al., 2011; for review see Bartels and Zeki, 2004; Acevedo, 2015), highlighting the role of attachment in sustained romantic love among newlywed pair-bonders. They are also consistent with research implicating *AVPR1a* in pair-bonding (Walum et al., 2008) and suggest the diversity of the pair-bonding system through its engagement of reward, memory, sensory, visual, and auditory functions.

### *OXTR* and Romantic Love Maintenance

The pattern of replicated interactions for *OXTR* rs53576 with romantic love maintenance were different from *AVPR1a* effects, appearing in the septum (bilaterally) and the left (L) VTA. Activation of L VTA has mostly appeared in studies of facial attractiveness, specifically showing response to smiling and supportive faces (Vrticka et al., 2008). Also, L VTA activation was shown in a study of males given intranasal OT in response to viewing facial images of their female partner (Scheele et al., 2013). Interestingly, when given OT males rated their partners as more attractive, but OT did *not* affect attractiveness ratings for a familiar matched control. These findings suggest that OT-related effects are partner-specific, thus facilitating attachment and pair-bond solidification. It should be noted that although we did not test for sex differences, sex may influence how OT affects mate choice and pair-bonding (Xu et al., 2020). Also, individual differences, such as personality and attachment style, may influence how OT interacts with pair-bonding choices (Pearce et al., 2019).

Activation of the septum—which is rich in binding sites for OT and, to a lesser extent, AVP—is consistent with animal studies showing that the septum is critical for pair-bond establishment (Liu et al., 2001). In humans, activation of the septum has been implicated in early-stage and long-term romantic love (Aron et al., 2005; Acevedo et al., 2011), and it was specifically associated with obsession-related items of the Passionate Love Scale (Hatfield and Sprecher, 1986) among long-term pair-bonders. We add to this body of work, showing OT’s effects in romantic love maintenance.

### Dopamine Gene Polymorphisms *(DRD4* and *COMT)* and Romantic Love Maintenance

Robust neural activations were positively correlated with romantic love maintenance and dopamine polymorphisms (*DRD4*-7R and *COMT* rs4680) in the L VTA/SN region and the posterior insular cortex at both time points. As noted above for the *OXTR* findings, the L VTA is specifically activated in response to facial attractiveness (e.g., Aron et al., 2005; Liang et al., 2010). Interestingly, the *DRD4*-7R genetic polymorphism, which is associated with reduced binding for dopamine and greater novelty seeking, was *negatively* correlated with romantic love scores at T1. Individuals with the 7R allele show higher rates of promiscuity and novelty seeking (He et al., 2018, meta-analysis; Munafo et al., 2008, meta-analysis; Garcia et al., 2010). Thus, it is not surprising that in the present study, individuals with the *DRD4*-7R variant showed lower romantic love scores but higher activation in the L VTA, where facial attractiveness promotes activation (Aharon et al., 2001; Winston et al., 2007). Dopamine-related gene expression (*COMT* and *DRD4*) in the L VTA suggests that facial attractiveness, reward, and more generally attraction mechanisms may be fruitful areas of examination for future research on sustaining romantic love in marriages.

*COMT* and *DRD4* also showed significant interactions in the insular cortex which is involved in a variety of functions including reward, emotion, social bonding, sensory processing, and self-awareness (for review see Gogolla, 2017). Specifically, the posterior insular cortex area where *DRD4*-effects were shown, is implicated in social support networks in elderly individuals (Cotton et al., 2019), making this an interesting region for future investigations of relationships. The human insula has also become a target for treatment in a variety of disorders such as substance abuse, depression, anxiety disorders, schizophrenia, and autism. Specifically, dopamine and opioid receptors in the insular cortex are thought to influence addiction (Ibrahim et al., 2019). Our findings highlight the role of attachment in sustained romantic love and are consistent with theories suggesting that romantic love is a “natural addiction” (Fischer et al., 2016).

Collectively, activation of the insula and other regions identified here (e.g., the STG, occipital area, hippocampus, PCL involved in sensory processing) are consistent with the idea that romantic love is an emergent property of pair-bonding whereby multi-sensory information is translated into processes such as communication, empathy, and decision-making as well as complex cognitive processes such as imagining a future together (Walum and Young, 2018). Thus, basic reward, sensory and higher-order cortical processes and their intersections, as exemplified herein, are critical for the maintenance of romantic love in established pair-bonds.

### Deactivations Associated With Romantic Love Maintenance

Deactivations emerged in association with romantic love maintenance, at both time points, in the inferior frontal gyrus (IFG) and the temporal gyrus. The IFG plays an evaluative role in multisensory stimuli and may be deactivated when evaluative processes are not engaged (Ethofer et al., 2006; Schirmer and Kotz, 2006). These results are consistent with previous brain imaging studies suggesting that in romantic love, suspension of negative judgment occurs, coinciding with deactivation in the temporal lobe (Zeki, 2007; Xu et al., 2011; Wang et al., 2020). IFG deactivation has also been associated with impairment in stopping a task once initiated (Chambers et al., 2006), consistent with the persistence of romantic love in the present sample.

### Future Directions and Limitations

Although this is the first study to provide evidence of the neural and genetic correlates of romantic love maintenance in a sample of newlyweds, it is important to recognize some limitations. The major limitation of this study is the small sample size. Although Friston et al. (2013) argued that small samples have advantages, small sample sizes and low statistical power may contribute to inflated effect sizes. Many of the effect sizes reported here were moderate to large, but with a larger sample in future studies effect sizes may be smaller. However, we relied on region-of-interest analyses and predicted/planned comparisons, which reduces possible statistical errors. Most importantly, many of our key findings were replicated. Replication is the most important statistical procedure for reliability of a result, and many of our key findings were shown at both time points. Also, a strength and a limitation of the study is the homogeneity of the sample.

Constraining the variables in the sample is important for the reproducibility of the results, but renders the results applicable to a limited population. Thus, generalizability of results is another limitation, as the participants in the present study were mostly well-adjusted, in-love, and highly satisfied with their relationship partner at T1. Nevertheless, this group of newlyweds experienced common marital concerns including the balancing of dual careers, managing domestic chores, and financial issues (Lavner and Bradbury, 2010). Also, in line with theories of “honeymoon effects,” one participant showed a steep decline in marital satisfaction and romantic love over the first year of marriage. Thus, it will be critical for future research to recruit larger and more diverse samples to capture the full range of relationship trajectories and pair-bonding strategies.

Another issue was that although we replicated many key findings at T1 and T2, other effects emerged separately for T1 and T2. For example, romantic love at T1 showed significant correlations in regions that are rich in serotonin (raphe and pons), while at T2 the patterns of neural activation were more robust in regions associated with emotion processing and rich in opioid receptors (amygdala and GP). Indeed, there were differences after 1 year of marriage that might indicate changes in attitude toward the partner, additional experiences with partner, envisioning a future together with children, conflict, and general life experiences. However, we refrained from speculating on what these differences in activation (and deactivation) might represent, but we did include the results so that future studies with larger samples (that may use different statistical approaches) may form hypotheses and determine if the neural mechanisms underlying romantic love, and its maintenance, change in consistent ways as a function of time.

To this point, it will be important for future studies on the biological basis of pair-bonding and romantic love to recruit couples with diverse levels of relationship quality. Relationship studies are often biased with positive couples because distressed/conflicted couples are more difficult to recruit as romantic partners often feel uncomfortable disclosing negative thoughts, sentiments, and doubts about their relationship. Additionally, social desirability effects may be especially strong around the time of the wedding; thus, appropriate measurement and objective markers are important for capturing couples that may be particularly vulnerable to conflict and sharp decreases in relationship quality.

In the current study we focused on four genes that have been implicated in social behaviors, including pair-bonding. There is a strong empirical basis for examining the particular genetic polymorphisms. For example, the dopamine receptor variant *DRD4*-7R, which we assessed in the present study, is associated with reproductive sexual behaviors (Eisenberg et al., 2007), desire for a wider variety of sexual behaviors (Halley et al., 2016), and higher rates of promiscuous behavior and infidelity (Garcia et al., 2010). However, there are other possibilities to explore. It will be critical for future research to examine a wider array of genetic polymorphisms underlying pair-bonding with larger samples, both with genome-wide association studies (GWAS) and more directed approaches with predicted polymorphisms. It has been shown that in many cases single genes have very small effect sizes (for review, see Fox and Beevers, 2016). However, GWAS studies are limited in that they require very large sample sizes (Landefeld et al., 2018). Other important genetic variants may also be examined in future studies, for example, the 5-HTTLPR VNTR of the serotonin transporter gene that has been associated with differences in life history strategy and risk-acceptance in mating competition (Minkov and Bond, 2015). Such findings are linked to a broader framework of life history theory than we investigated here, but they are relevant to variation in human mating and pair-bonding strategies (Minkov and Bond, 2015; Pearce et al., 2019).

Also, it is important to note that although identifying biological markers for pair-bonding in group studies is helpful, individual differences must be accounted for. For example, in recent years OT has received significant attention for strengthening pair-bonds (e.g., Quintana et al., 2019). However, responses to OT may vary according to some oxytocin genetic polymorphisms and gender (e.g., Pearce et al., 2019; Xu et al., 2020). Also, results from brain imaging studies indicate that oxytocin genetic variants may influence couples’ sociosexual feelings, sexual behaviors, and intimacy (e.g., Acevedo et al., 2019a, b; Pearce et al., 2019).

Finally, although this study is the first to report neural and genetic mechanisms underlying changes in romantic love in first-time newlyweds, it would be beneficial for future neurobiological studies to expand measurements beyond the first year of marriage. This might capture important changes that occur over marital development such as the addition of offspring, career transitions, and increased interdependence that is an inevitable aspect of marriage.

## Conclusion

Romantic love plays a critical role in relationship initiation, longevity, and individual well-being. However, the biological mechanisms underlying romantic love maintenance in marriages have gone largely unexplored. For the first time, we investigated anatomically specific neural activations together with targeted genetic variants (*AVPR1a* rs3, *OXTR* rs53576, *DRD4*-7R, and *COMT* rs4680) to determine if these polymorphisms are associated with romantic love maintenance among newlyweds. Our results show that romantic love may be sustained via genetically influenced processes in widespread reward, emotion, and primary sensory regions of the human brain. Taken together, these findings suggest an important role for mammalian attachment and reward mechanisms in generating high-quality pair-bonds resilient to declines in romantic love over time. In addition, the current study provides initial evidence of how genetic polymorphisms mediate variability in behaviors related to romantic love maintenance and pair-bonding during the first year of marriage. Finally, the results are consistent with the overall hypothesis that romantic love is part of a suite of human reproductive strategies, particularly long-term ones, and a developed form of a mammalian drive to pursue and keep preferred mates. This view, along with these findings about genetic variability, can be therapeutically useful by placing romantic love and its maintenance in a larger context than the individual couple seeking help.

## Author’s Note

The data utilized for the current study were part of a broader project (see Acevedo et al., 2014, 2019a,2019b for other papers), and these results have been published elsewhere (Acevedo et al., 2014, 2019a,2019b). No analyses in the present research are redundant with any published findings.

## Data Availability Statement

The raw data supporting the conclusions of this article will be made available by the authors, without undue reservation, to any qualified researcher.

## Ethics Statement

The studies involving human participants were reviewed and approved by IRBs of the University of California, Santa Barbara and Einstein College of Medicine. The patients/participants provided their written informed consent to participate in this study.

## Author Contributions

All authors listed have made a substantial, direct and intellectual contribution to the work, and approved it for publication.

## Conflict of Interest

The authors declare that the research was conducted in the absence of any commercial or financial relationships that could be construed as a potential conflict of interest.

## Footnotes

1 The VTA/SN activation for the *OXTR* rs53576 interaction at T2 was only marginally significant at *p* = 0.09. However, given the strong theoretical and empirical basis for its examination, we noted the VTA replication for Times 1 and 2 in this sample.

## References

[B1] Acevedo B. Aron A. Fisher H. Brown L. (2012). Neural correlates of marital satisfaction and well- being: reward, empathy, and affect. *J. Clin. Neuropsychiatr.* 9 20–31.

[B2] Acevedo B. Aron E. Aron A. Sangster M. Collins N. Brown L. (2014). The highly sensitive brain: an fMRI study of sensory processing sensitivity. *Brain Behav.* 4 580–594. 10.1002/brb3.242 25161824 PMC4086365

[B3] Acevedo B. Geher G. Poulin M. Grafton S. Brown L. (2019a). The neural and genetic correlates of satisfying sexual activity in heterosexual pair-bonds. *Brain Behav.* 9 1–32. 10.1002/brb3.1289 31090198 PMC6576152

[B4] Acevedo B. Poulin M. Brown L. (2019b). Beyond romance: neural and genetic correlates of altruism in pair-bonds. *Behav. Neurosci.* 133 18–31. 10.1037/bne0000293 30688485

[B5] Acevedo B. P. (2015). “Neural correlates of human attachment: evidence from fMRI studies of adult pair-bonding,” in *Bases of Adult Attachment. Linking Brain, Mind And Behavior*, eds Zayas V. Hazan C. (New York, NY: Springer), 185–194. 10.1007/978-1-4614-9622-9_9

[B6] Acevedo B. P. Aron A. (2009). Does a long-term relationship kill romantic love? *Rev. Gen. Psychol.* 13 59–65. 10.1037/a0014226

[B7] Acevedo B. P. Aron A. Fisher H. E. Brown L. L. (2011). Neural correlates of long-term intense romantic love. *Soc. Cogn. Affect. Neurosci.* 7 145–159. 10.1093/scan/nsq092 21208991 PMC3277362

[B8] Aharon I. Etcoff N. Ariely D. Chabris C. F. O’Connor E. Breiter H. C. (2001). Beautiful faces have variable reward value: fMRI and behavioral evidence. *Neuron* 32 537–531. 10.1016/S0896-6273(01)00491-3 11709163

[B9] Alger S. J. Kelm-Nelson C. A. Stevenson S. A. Juang C. Gammie S. C. Riters L. V. (2020). Complex patterns of dopamine-related gene expression in the ventral tegmental area of male zebra finches relate to dyadic interactions with long-term female partners. *Genes Brain Behav.* 19 1–13. 10.1111/gbb.12619 31634415 PMC7582019

[B10] Aron A. Fisher H. Mashek D. J. Strong G. Li H. Brown L. L. (2005). Reward, motivation, and emotion systems associated with early-stage intense romantic love. *J. Neurophysiol.* 94 327–337. 10.1152/jn.00838.2004 15928068

[B11] Asghari V. Sanyal S. Buchwaldt S. Paterson A. Jovanovic V. Van H. H. (1995). Modulation n f intracellular cyclic AMP levels by different human Dopamine D4 receptor variants. *J. Neurochem.* 65 1157–1165. 10.1046/j.1471-4159.1995.65031157.x 7643093

[B12] Bancroft T. D. Hockley W. E. Servos P. (2014). Does stimulus complexity determine whether working memory storage relies on prefrontal or sensory cortex? *Atten. Percept. Psychophys.* 76 1954–1961. 10.3758/s13414-013-0604-0 24452382

[B13] Bartels A. Zeki S. (2000). The neural basis of romantic love. *Neuroreport* 11 3829–3834. 10.1097/00001756-200011270-00046 11117499

[B14] Bartels A. Zeki S. (2004). The neural correlates of maternal and romantic love. *Neuroimage* 21 1155–1166. 10.1016/j.neuroimage.2003.11.003 15006682

[B15] Batson C. D. (2011). What’s wrong with morality? *Emot. Rev.* 3 230–236. 10.1207/s15327957pspr0301_3 15647147

[B16] Baumeister R. F. Bratslavsky E. (1999). Passion, intimacy, and time: passionate love as a function of change in intimacy. *Pers. Soc. Psychol. Rev.* 3 49–67. 10.1207/s15327957pspr0301_3 15647147

[B17] Baumeister R. F. Leary M. R. (1995). The need to belong: desire for interpersonal attachments as a fundamental human motivation. *Psychol. Bull.* 117 497–529. 10.1037/0033-2909.117.3.497 7777651

[B18] Berridge K. C. Robinson T. E. (2003). Parsing reward. *Trends Neurosci.* 26 507–513. 10.1016/S0166-2236(03)00233-9 12948663

[B19] Berscheid E. Hatfield E. (1969). *Interpersonal Attraction.* New York, NY: Addison-Wesley.

[B20] Birnbaum J. Flemming S. Reichard N. Soares A. B. Mesen-Ramirez P. Jonscher E. (2017). A genetic system to study Plasmodium falciparum protein function. *Nat. Methods* 14 450–456. 10.1038/nmeth.4223 28288121

[B21] Borrow A. Cameron A. (2011). The role of oxytocin in mating and pregnancy. *Horm. Behav.* 61 266–276. 10.1016/j.yhbeh.2011.11.001 22107910

[B22] Bradbury T. (ed.) (1998). *The Developmental Course of Marital Dysfunction.* Cambridge: Cambridge University Press.

[B23] Brunnlieb C. Nave G. Camerer C. F. Schosser S. Vogt B. Münte T. F. (2016). Vasopressin increases human risky cooperative behavior. *Proc. Natl. Acad. Sci. U.S.A.* 113 2051–2056. 10.1073/pnas.1518825113 26858433 PMC4776476

[B24] Buffone A. E. K. Poulin M. J. (2014). Empathy, target distress, and neurohormone genes interact to predict aggression for others-even without provocation. *Pers. Soc. Psychol. Bull.* 40 1406–1422. 10.1177/0146167214549320 25287464

[B25] Buss D. M. (2018). “The evolution of love in humans,” in *The New Psychology of Love*, eds Sternberg R. Sternberg K. (Cambridge: Cambridge University Press), 42–63. 10.1017/9781108658225.004

[B26] Chambers C. D. Bellgrove M. A. Stokes M. G. Henderson T. R. Garavan H. Robertson I. H. (2006). Executive “brake failure” following deactivation of human frontal lobe. *J. Cogn. Neurosci.* 18 444–455. 10.1162/jocn.2006.18.3.444 16513008

[B27] Chen J. Lipska B. K. Halim N. Ma Q. D. Matsumoto M. Melhem S. (2004). Functional analysis of genetic variation in catechol-O-methyltransferase COMT: effects on mRNA, protein, and enzyme activity in postmortem human brain. *Am. J. Hum. Genet.* 75 807–821. 10.1086/425589 15457404 PMC1182110

[B28] Childress A. R. Ehrman R. N. Wang Z. Li Y. Sciortino N. Hakun J. (2008). Prelude to passion: Limbic activation by “unseen” drug and sexual cues. *PLoS One* 3:e1506. 10.1371/journal.pone.0001506 18231593 PMC2204052

[B29] Cornwell R. E. Law Smith M. J. Boothroyd L. G. Moore F. R. Davis H. P. Stirrat M. (2006). Reproductive strategy, sexual development and attraction to facial characteristics. *Phils. Trans. B Soc. Lond. B Biol. Sci.* 361 2153–2154. 10.1098/rstb.2006.1936 17118929 PMC1764838

[B30] Cotton K. Verghese J. Blumen H. M. (2019). Gray matter volume covariance networks, social support and cognition in older adults. *J. Gerontol. Ser. B* gbz023. 10.1093/geronb/gbz023 30816944 PMC7265803

[B31] D’Ardenne K. McClure S. M. Nystrom L. E. Cohen J. D. (2008). BOLD responses reflecting dopaminergic signals in the human ventral tegmental area. *Science* 28 1264–1267. 10.1126/science.1150605 18309087

[B32] de Waal F. B. (2008). Putting the altruism back into altruism: the evolution of empathy. *Annu. Rev. Psychol.* 59 279–300. 10.1146/annurev.psych.59.103006.093625 17550343

[B33] de Waal F. B. M. Gavrilets S. (2013). Monogamy with a purpose. *PNAS* 110 15167–15168. 10.1073/pnas.1315839110 24046334 PMC3780882

[B34] Del Giudice M. Gangestad S. W. Kaplan H. S. (2015). “Life history theory and evolutionary psychology,” in *The Handbook of Evolutionary Psychology*, 2nd Edn, ed. Buss D. M. (New York: Wiley Online Library), 88–114. 10.1002/9781119125563.evpsych102

[B35] Denys D. van der Wee N. Janssen J. De Geus F. Westenberg H. G. (2004). Low level of dopaminergic D2 receptor binding in obsessive-compulsive disorder. *Biol. Psychiatry* 55 1041–1045. 10.1016/j.biopsych.2004.01.023 15121489

[B36] Eisenberg D. Gollust S. E. Golberstein E. Hefner J. L. (2007). Prevalence and correlates of depression, anxiety, and suicidality among university students. *Am. J. Orthopsychiatry* 77 534–542. 10.1037/0002-9432.77.4.534 18194033

[B37] Ethofer T. Anders S. Erb M. Droll C. Royen L. Saur R. (2006). Impact of voice on emotional judgment of faces: an event-related fMRI study. *Hum. Brain Mapp.* 27 707–714. 10.1002/hbm.20212 16411179 PMC6871326

[B38] Fields H. L. Hjelmstad G. O. Margolis E. B. Nicola S. M. (2007). Ventral tegmental area neurons in learned appetitive behavior and positive reinforcement. *Annu. Rev. Neurosci.* 30 289–316. 10.1146/annurev.neuro.30.051606.094341 17376009

[B39] Fischer H. E. Xu X. Aron A. Brown L. L. (2016). Intense, passionate, romantic love: a natural addiction? How the fields that investigate romance and substance abuse can inform each other. *Front. Psychol.* 7:687. 10.3389/fpsyg.2016.00687 27242601 PMC4861725

[B40] Fisher H. E. Aron A. Brown L. L. (2006). Romantic love: a mammalian brain system for mate choice. *Philos. Trans. R. Soc. Lond. B Biol. Sci.* 361 2173–2186. 10.1098/rstb.2006.1938 17118931 PMC1764845

[B41] Fletcher G. J. Simpson J. A. Campbell L. Overall N. C. (2015). Pair-bonding, romantic love, and evolution: the curious case of Homo sapiens. *Perspect. Psychol. Sci.* 10 20–36. 10.1177/1745691614561683 25910380

[B42] Fox E. Beevers C. G. (2016). Differential sensitivity to the environment: contribution of cognitive biases and genes to psychological wellbeing. *Mol. Psychiatry* 21 1657–1662. 10.1038/mp.2016.114 27431291 PMC5075581

[B43] Frascella J. Potenza M. N. Brown L. L. Childress A. R. (2010). Shared brain vulnerabilities open the way for nonsubstance addictions: caving addiction at a new joint? *Ann. N. Y. Acad. Sci.* 1187 294–315. 10.1111/j.1749-6632.2009.05420.x 20201859 PMC3671907

[B44] Friston K. Schwartenbeck P. FitzGerald T. Moutoussis M. Behrens T. Raymond R. J. (2013). The anatomy of choice: active inference and agency. *Front. Hum. Neurosci.* 7:598. 10.3389/fnhum.2013.00598 24093015 PMC3782702

[B45] Galak J. Redden J. P. (2018). The properties and antecedents of hedonic decline. *Annu. Rev. Psychol.* 69 1–25. 10.1146/annurev-psych-122216-011542 28854001

[B46] Galvez-Pol A. Calvo-Merino B. Capilla A. Forster B. (2018). Persistent recruitment of somatosensory cortex during active maintenance of hand images in working memory. *Neuroimage* 174 153–163. 10.1016/j.neuroimage.2018.03.024 29548846

[B47] Garcia J. R. MacKillop J. Aller E. L. Merriwether A. M. Wilson D. S. Lum J. K. (2010). Associations between Dopamine D4 Receptor gene variation with both infidelity and sexual promiscuity. *PLoS One* 5:e14162. 10.1371/journal.pone.0014162 21152404 PMC2994774

[B48] Gard D. E. Germans Gard M. Kring A. M. John O. P. (2006). Anticipatory and consummatory components of the experience of pleasure: a scale development study. *J. Res. Pers.* 40 1086–1102. 10.1002/pchj.207 29431282

[B49] Genovese C. R. Lazar N. A. Nichols T. (2002). Thresholding of statistical maps in functional neuroimaging using the false discovery rate. *Neuroimage* 15 870–878. 10.1006/nimg.2001.1037 11906227

[B50] Georgiadis J. R. Farrell M. J. Boessen R. Denton D. A. Gavrilescu M. Kortekaas R. (2010). Dynamic subcortical blood flow during male sexual activity with ecological validity: a perfusion fMRI study. *Neuroimage* 50 208–216. 10.1016/j.neuroimage.2009.12.034 20006720

[B51] Getz L. L. Carter S. Gavish L. (1981). The mating system of the prairie vole, Michrotus ochrogaster: field and laboratory evidence for pair-bonding. *Behav. Ecol. Sociobiol.* 8 189–194. 10.1007/BF00299829

[B52] Gogolla N. (2017). The insular cortex. *Curr. Biol.* 27 580–586. 10.1016/j.cub.2017.05.010 28633023

[B53] Gong P. Fan H. Liu J. Yang X. Zhang K. Zhou X. (2017). Revisiting the impact of OXTR rs53576 on empathy: a population-based study and a meta-analysis. *Psychoneuroendocrinology* 80 131–136. 10.1016/j.psyneuen.2017.03.005 28343138

[B54] Goodman W. K. McDougle C. J. Price L. H. Riddle M. A. Pauls D. L. Leckman A. (1990). Beyond the serotonin hypothesis: a role for dopamine in some forms of obsessive compulsive disorder? *J. Clin. Psychiatry* 51 36–43. 2199433

[B55] Gottman J. M. Coan J. Carrere S. Swanson C. (1998). Predicting marital happiness and stability from newlywed interactions. *J. Marriage Fam.* 60 5 10.2307/353438

[B56] Grewen K. M. Girdler S. S. Amico J. Light K. C. (2005). Effects of partner support on resting oxytocin, cortisol, norepinephrine, and blood pressure before and after warm partner contact. *Psychosom. Med.* 67 531–538. 10.1097/01.psy.0000170341.88395.47 16046364

[B57] Halley A. C. Boretsky M. Puts D. A. Shriver M. (2016). Self-reported sexual behavioral interests and polymorphisms in the dopamine receptor D4 (DRD4) Exon III VNTR in heterosexual young adults. *Arch. Sex. Behav.* 45 2091–2100. 26581567 10.1007/s10508-015-0646-6

[B58] Hatfield E. Sprecher S. (1986). Measuring passionate love in intimate relationships. *J. Adolesc.* 9 383–410. 10.1016/S0140-1971(86)80043-4 3805440

[B59] Hatfield E. C. Pillemer J. T. O’Brien M. U. Le Y.-C. L. (2008). The endurance of love: passionate and companionate love in newlywed and long-erm marriages. *Interpersona* 2 35–64. 10.1016/S0140-1971(86)80043-4

[B60] He Y. Martin N. Zhu G. Liu Y. (2018). Candidate genes for novelty-seeking: a meta-analysis of association studies of DRD4 exon III and COMT Val158Met. *Psychiatr. Genet.* 28 97–109. 10.1097/YPG.0000000000000209 30260901

[B61] Heleven E. Van Overwalle F. (2016). The person within: memory codes for persons and traits using fMRI repetition suppression. *Soc. Cogn. Affect. Neurosci.* 11 159–171. 10.1093/scan/nsv100 26371337 PMC4692324

[B62] Hendrick C. Hendrick S. (1986). A theory and method of love. *J. Pers. Soc. Psychol.* 50 392–402. 10.1037/0022-3514.50.2.392

[B63] Hendrick S. S. (1988). A generic measure of relationship satisfaction. *J. Marriage Fam.* 50 93–98. 10.2307/352430

[B64] Huston T. L. Caughlin J. P. Houts R. M. Smith S. E. George L. J. (2001). The connubial crucible. Newlywed years as predictors of marital delight, distress, and divorce. *J. Pers. Soc. Psychol.* 80 237–252. 10.1037/0022-3514.80.2.237 11220443

[B65] Huston T. L. Houts R. M. (1998). “The psychological infrastructure of courtship and marriage: the role of personality and compatibility in romantic relationships,” in *The Developmental Course of Marital Dysfunction*, ed. Bradbury T. N. (New York, NY: Cambridge University Press), 114–151. 10.1017/CBO9780511527814.006

[B66] Ibrahim C. Rubin-Kahana D. S. Pushparaj A. Musiol M. Blumberger D. M. Daskalakis Z. J. (2019). The Insula: a brain stimulation target for the treatment of addiction. *Front. Pharmacol.* 10:720. 10.3389/fphar.2019.00720 31312138 PMC6614510

[B67] Ikemoto S. Yang C. Tan A. (2015). Basal ganglia circuit loops, dopamine and motivation: a review and enquiry. *Behav. Brain Res.* 290 17–31. 10.1016/j.bbr.2015.04.018 25907747 PMC4447603

[B68] Insel T. R. Wang Z. X. Ferris C. F. (1994). Patterns of brain vasopressin receptor distribution associated with social organization in microtine rodents. *J. Neurosci.* 14 5381–5392. 10.1523/JNEUROSCI.14-09-05381.1994 8083743 PMC6577077

[B69] Jacobs Bao K. Lyubomirsky S. (2013). Making it last: combating hedonic adaptation in romantic relationships. *J. Posit. Psychol.* 8 196–206. 10.1080/17439760.2013.777765

[B70] Jarcho J. M. Berkman E. T. Lieberman M. D. (2011). The neural basis of rationalization: cognitive dissonance reduction during decision-making. *Soc. Cogn. Affect. Neurosci.* 6 460–467. 10.1093/scan/nsq054 20621961 PMC3150852

[B71] Kamp Dush C. M. Taylor M. G. Kroeger R. A. (2008). Marital happiness and psychological well-being across the life course. *Fam. Relat.* 57 211–226. 10.1111/j.1741-3729.2008.00495.x 23667284 PMC3650717

[B72] Katz A. C. Sarapas C. Bishop J. R. Patel S. R. Shankman S. A. (2015). The mediating effect of prefrontal asymmetry on the relationship between the COMT Val158 Met SNP and trait consummatory positive affect. *Cogn. Emot.* 29 867–881. 10.1080/02699931.2014.951030 25195915 PMC4362801

[B73] Kim Y. J. Khoshkhoo S. Frankowski J. C. Zhu B. Abbasi S. Lee S. (2018). Chd2 is necessary for neural circuit development and long-term memory. *Neuron* 100 1180–1193. 10.1016/j.neuron.2018.09.049 30344048 PMC6479120

[B74] Knafo A. Zahn-Waxler C. van Hulle C. Robinson J. L. Rhee S. H. (2008). The developmental origins of a disposition toward empathy: genetic and environmental contributions. *Emotion* 8 737–752. 10.1037/a0014179 19102585

[B75] Kragel P. A. LaBar K. S. (2016). Somatosensory representations link the perception of emotional expressions and sensory experience. *eNeuro* 3:ENEURO.0090-15. 10.1523/ENEURO.0090-15.2016 27280154 PMC4894916

[B76] Krebs R. M. Boehler C. N. Egner T. Woldorff M. G. (2011). The neural underpinnings of how reward associations can both guide and misguide attention. *J. Neurosci.* 31 9752–9759. 10.1523/JNEUROSCI.0732-11.2011 21715640 PMC3142621

[B77] Lancaster T. M. Linden D. E. Heerey E. A. (2012). COMT val158met predicts reward responsiveness in humans. *Genes Brain Behav.* 11 986–992. 10.1111/j.1601-183X.2012.00838.x 22900954

[B78] Landefeld C. C. Hodgkinson C. A. Spagnolo P. A. Marietta C. A. Shen P. H. Sun H. (2018). Effects on gene expression and behavior of untagged short tandem repeats: the case of arginine vasopressin receptor 1a (AVPR1a) and externalizing behaviors. *Transl. Psychiatry* 8:72. 10.1038/s41398-018-0120-z 29581423 PMC5913313

[B79] Lavner J. A. Bradbury T. N. (2010). Patterns of change in marital satisfaction over the newlywed years. *J. Marriage Fam.* 72:5. 10.1111/j.1741-3737.2010.00757.x 21116452 PMC2992446

[B80] Levenson R. W. Carstensen L. L. Gottman J. M. (1993). Long-term marriage: age, gender, and satisfaction. *Psychol. Aging* 8 301–313. 10.1037/0882-7974.8.2.301 8323733

[B81] Li J. Zhao Y. Li R. Broster L. S. Zhou C. Yang S. (2015). Association of oxytocin receptor gene (OXTR) rs53576 polymorphism with sociality: a meta-analysis. *PLoS One* 10:e0131820. 10.1371/journal.pone.0131820 26121678 PMC4488068

[B82] Liang X. Zebrowitz L. A. Zhang Y. (2010). Neural activation in the ‘reward circuit’. shows a nonlinear response to facial attractiveness. *Soc. Neurosci.* 5 320–334. 10.1080/17470911003619916 20221946 PMC2885490

[B83] Light K. Grewen K. M. Amico J. A. (2005). More frequent partner hugs and higher oxytocin levels are linked to lower blood pressure and heart rate in premenopausal women. *Biol. Psychol.* 69 5–21. 10.1016/j.biopsycho.2004.11.002 15740822

[B84] Lim M. M. Wang Z. Olazabal D. E. Ren X. Terwilliger E. F. Young L. J. (2004). Enhanced partner preference in a promiscuous species by manipulating the expression of a single gene. *Nature* 429 754–757. 10.1038/nature02539 15201909

[B85] Lim M. M. Young L. J. (2004). Vasopressin-dependent neural circuits underlying pair bond formation in the monogamous prairie vole. *Neuroscience* 125 35–45. 10.1016/j.neuroscience.2003.12.008 15051143

[B86] Lim T. Y. Al-Betar M. A. Khader A. T. (2015). Adaptive pair bonds in genetic algorithm: an application to real-parameter optimization. *Appl. Math. Comput.* 252 503–519. 10.1016/j.amc.2014.12.030

[B87] Liu Y. Curtis J. T. Wang Z. (2001). Vasopressin in the lateral septum regulates pair bond formation in male prairie voles (*Microtus ochrogaster*). *Behav. Neurosci.* 115 910–919. 10.1037/0735-7044.115.4.910 11508730

[B88] Lorber M. F. Erlanger A. C. E. Heyman R. E. O’Leary K. D. (2015). The honeymoon effect: does it exist and can it be predicted? *Prevent. Sci.* 16 550–559. 10.1007/s11121-014-0480-4 24643282

[B89] Lukas D. Clutton-Brock T. H. (2013). The evolution of social monogamy in mammals. *Science* 341 526–530. 10.1126/science.1238677 23896459

[B90] Mai J. K. Matjtanik M. Paxinos G. (2016). *Atlas of the Human Brain*, 4th Edn San Diego: Academic Press.

[B91] Maner J. K. Gailliot M. T. Miller S. L. (2009). The implicit cognition of relationship maintenance. Inattention to attractive alternatives. *J. Exp. Soc. Psychol.* 45 174–179. 10.1016/j.jesp.2008.08.002

[B92] Männistö P. T. Kaakkola S. (1999). Catechol-O-methyltransferase (COMT): biochemistry, molecular biology, pharmacology, and clinical efficacy of the new selective COMT inhibitors. *Pharmacol Rev.* 51 593–628. 10581325

[B93] Mashek D. Aron A. Fisher H. E. (2000). Identifying, evoking, and measuring intense feelings of romantic love. *Represent. Res. Soc. Psychol.* 24 48–55.

[B94] McNulty J. K. Olson M. A. Meltzer A. L. Shaffer M. J. (2013). Though they may be unaware, newlyweds implicitly know whether their marriage will be satisfying. *Science* 342 1119–1120. 10.1126/science.1243140 24288337

[B95] Mendoza S. P. Mason W. A. (1986). Parental division of labour and differentiation of attachments in a monogamous primate (*Callicebus moloch*). *Anim. Behav.* 34 1336–1347. 10.1016/S0003-3472(86)80205-6

[B96] Meyer-Lindenberg A. (2008). Psychology. Trust me on this. *Science* 321 778–780. 10.1126/science.1162908 18687945

[B97] Miller P. J. E. Niehuis S. Huston T. L. (2006). Positive illusions in marital relationships: a 13-year longitudinal study. *Pers. Soc. Psychol. Bull.* 32 1579–1594. 10.1177/0146167206292691 17122172

[B98] Minkov M. Bond M. H. (2015). Genetic polymorphisms predict national differences in life history strategy and time orientation. *Pers. Individ. Differ.* 76 204–215. 10.1016/j.paid.2014.12.014

[B99] Muckli L. Petro L. S. (2017). The significance of memory in sensory cortex. *Trends Neurosci.* 40 255–256. 10.1016/j.tins.2017.03.004 28363477 PMC5421742

[B100] Munafo M. R. Yalcin B. Willis-Owen S. A. Flint J. (2008). Association of the dopamine D4 receptor (DRD4) gene and approach-related personality traits: meta-analysis and new data. *Biol. Psychiatry* 63 119–206. 10.1016/j.biopsych.2007.04.006 17574217

[B101] Nagy K. Greenlee M. W. Kovacs G. (2012). The lateral occipital cortex in the face perception network: an effective connectivity study. *Front. Psychol.* 3:141. 10.3389/fpsyg.2012.00141 22593748 PMC3349303

[B102] Niehuis J. B. Owen J. Valentine J. C. Black S. W. Halford T. C. Parazak S. E. (2018). Therapeutic alliance, empathy, and genuineness in individual adult psychotherapy: a meta-analytic review. *Psychotherapy Res.* 28 1–13. 10.1080/10503307.2016.1204023 27389666

[B103] Niehuis S. Lee K.-H. Reifman A. Swenson A. Hunsaker S. (2011). Idealization and disillusionment in intimate relationships: a review of theory, method, and research. *J. Fam. Theor. Rev.* 3 273–302. 10.1111/j.1756-2589.2011.00100.x

[B104] Noori H. R. Cosa Linan A. Spanagel R. (2016). Largely overlapping neuronal substrates of reactivity to drug, gambling, food and sexual cues: a comprehensive meta-analysis. *Eur. Neuropsychopharmacol.* 26 1419–1430. 10.1016/j.euroneuro.2016.06.013 27397863

[B105] Nowak N. T. Weisfeld G. E. Imamoðlu O. Weisfeld C. Butovskaya M. Shen J. (2014). Attractiveness and spousal infidelity as predictors of sexual fulfillment without the marriage partner in couples from five cultures. *Hum. Ethol. Bull.* 291 18–38.

[B106] O’Leary K. D. Acevedo B. P. Aron A. Huddy L. Mashek D. (2012). Is long-term love more than a rare phenomenon? If so, what are its correlates? *Soc. Psychol. Pers. Sci.* 3 241–249. 10.1177/1948550611417015

[B107] Ortigue S. Bianchi-Demicheli F. Hamilton A. F. D. C. Grafton S. T. (2007). The neural basis of love as a subliminal prime: an event-related functional magnetic resonance imaging study. *J. Cogn. Neurosci.* 19 1218–1230. 10.1162/jocn.2007.19.7.1218 17583996

[B108] Ortigue S. Bianchi-Demicheli F. Patel N. Frum C. Lewis J. W. (2010). Neuroimaging of love: fMRI meta-analysis evidence toward new perspectives in sexual medicine. *J. Sex. Med.* 7 3541–3552. 10.1111/j.1743-6109.2010.01999.x 20807326

[B109] Pearce E. Wlodarski R. Machin A. Dunbar R. I. (2019). Genetic influences on social relationships: sex differences in the mediating role of personality and social cognition. *Adapt. Hum. Behav. Physiol.* 5 331–351. 10.1007/s40750-019-00120-5

[B110] Phan K. L. Wager T. Taylor S. F. Liberzon I. (2002). Functional neuroanatomy of emotion: a meta-analysis of emotion activation studies in PET and fMRI. *Neuroimage* 16 331–348. 10.1006/nimg.2002.1087 12030820

[B111] Poulin M. J. Holman E. A. Buffone A. (2012). The neurogenetics of nice: receptor genes for oxytocin and vasopressin interact with threat to predict prosocial behavior. *Psychol. Sci.* 23 446–452. 10.1177/0956797611428471 22457427

[B112] Quintana D. S. Rokicki J. van der Meer D. Alnæs D. Kaufmann T. Córdova-Palomera A. (2019). Oxytocin pathway gene networks in the human brain. *Nat. Commun.* 10:668. 10.1038/s41467-019-08503-8 30737392 PMC6368605

[B113] Raghanti M. A. Edler M. K. Stephenson A. R. Munger E. L. Jacobs B. Hof P. R. (2018). A neurochemical hypothesis for the origin of hominids. *PNAS* 115:E1108–E1116. 10.1073/pnas.1719666115 29358369 PMC5819450

[B114] Risinger R. C. Salmeron B. J. Ross T. J. Amen S. L. Sanfilipo M. Hoffmann R. G. (2005). Neural correlates of high and craving during cocaine self-administration using BOLD fMRI. *Neuroimage* 26 1097–1108. 10.1016/j.neuroimage.2005.03.030 15886020

[B115] Rodrigues S. M. Saslow L. R. Garcia N. John O. P. Keltner D. (2009). Oxytocin receptor genetic variation relates to empathy and stress reactivity in humans. *Proc. Natl. Acad. Sci. U.S.A.* 106 437–21441. 10.1073/pnas.0909579106 19934046 PMC2795557

[B116] Schacht R. Kramer K. L. (2019). Are we monogamous? A review of the evolution of pair-bonding in humans and its contemporary variation cross-culturally. *Front. Ecol. Evol.* 7:230 10.3389/fevo.2019.00230

[B117] Scheele D. Wille A. Kendrick K. M. Stoffel-Wagner B. Becker B. Güntürkün O. (2013). Oxytocin enhances brain reward system responses in men viewing the face of their female partner. *PNAS* 110 20308–20313. 10.1073/pnas.1314190110 24277856 PMC3864312

[B118] Schirmer A. Kotz S. A. (2006). Beyond the right hemisphere: brain mechanisms mediating vocal emotional processing. *Trends Cogn. Sci.* 10 24–30. 10.1016/j.tics.2005.11.009 16321562

[B119] Schultz R. T. Grelotti D. J. Klin A. Kleinman J. Van der Gaag C. Marois R. (2003). The role of the fusiform face area in social cognition: implications for the pathobiology of autism. *Philos. Trans. R. Soc. Lond. B Biol. Sci.* 358 415–427. 10.1098/rstb.2002.1208 12639338 PMC1693125

[B120] Schultz W. (2010). Dopamine signals for reward value and risk: basic and recent data. *Behav. Brain Funct.* 6:24. 10.1186/1744-9081-6-24 20416052 PMC2876988

[B121] Shahrazad W. W. S. Mohd S. Chond S. T. (2012). Investigating the factor structure of the Love Attitude Scale (LAS) with Malaysian samples. *Asian Soc. Sci.* 8 66–73. 10.5539/ass.v8n9p66

[B122] Striepens N. Kendrink K. M. Maier W. Hurlemann R. (2011). Prosocial effects of oxytocin and clinical evidence for its therapeutic potential. *Front. Neuroendocrinol.* 32 426–450. 10.1016/j.yfrne.2011.07.001 21802441

[B123] Takahashi K. Mizuno K. Sasaki A. T. Wada Y. Tanaka M. Ishii A. (2015). Imaging the passionate stage of romantic love by dopamine dynamics. *Front. Hum. Neurosci.* 9:191. 10.3389/fnhum.2015.00191 25914637 PMC4391262

[B124] Taylor S. (2016). Disorder-specific genetic factors in obsessive-compulsive disorder: a comprehensive meta-analysis. *Am. J. Med. Genet. B Neuropsychiatr. Genet.* 171 325–332. 10.1002/ajmg.b.32407 26616111

[B125] Tennov D. (1999). *Love and Limerence: The Experience of Being In Love.* Archdale, NC: Scarbough House.

[B126] Thornton M. A. Mitchell J. P. (2018). Theories of person perception predict patterns of neural activity during mentalizing. *Cereb. Cortex* 28 3505–3520. 10.1093/cercor/bhx216 28968854

[B127] Uzefovsky F. Döring A. K. Knafo-Noam A. (2015). Values in middle childhood. social and genetic contributions. *Soc. Dev.* 25 482–502. 10.1111/sode.12155

[B128] van Dijk W. W. Zeelenberg M. (2002). What do we talk about when we talk about disappointment? Distinguishing outcome-related disappointment from person-related disappointment. *Cogn. Emot.* 16 787–807. 10.1080/02699930143000563

[B129] Vrticka P. Andersson F. Grandjean D. Sander D. Vuilleumier P. (2008). Individual attachment style modulates human amygdala and striatum activation during social appraisal. *PLoS One* 3:e2868. 10.1371/journal.pone.0002868 18682729 PMC2478709

[B130] Wager T. D. Phan K. L. Liberzon I. Taylor S. F. (2003). Valence, gender, and lateralization of functional brain anatomy in emotion: a meta-analysis of findings from neuroimaging. *Neuroimage* 19 513–531. 10.1016/S1053-8119(03)00078-8 12880784

[B131] Walum H. Westberg L. Henningsson S. Neiderhiser J. M. Reiss D. Igl W. (2008). Genetic variation in the vasopressin receptor 1a gene (AVPR1A) associates with pair-bonding behavior in humans. *PNAS* 105 14153–14156. 10.1073/pnas.0803081105 18765804 PMC2533683

[B132] Walum H. Young L. J. (2018). The neural mechanisms and circuitry of the pair bond. Nature reviews. *Neuroscience* 19 643–654. 10.1038/s41583-018-0072-6 30301953 PMC6283620

[B133] Wang C. Song S. Uquillas F. D. O. Zilverstand A. Song H. Chen H. (2020). Altered brain network organization in romantic love as measured with resting-state fMRI and graph theory. *Brain Imaging Behav.* 1–14. 10.1007/s11682-019-00226-0 31898089

[B134] Wichers M. C. Myin-Germeys I. Jacobs N. Peeters F. Kenis G. Derom C. (2007). Evidence that moment-to-moment variation in positive emotions buffer genetic risk for depression: a momentary assessment twin study. *Acta Psychiatr. Scand.* 115 451–457. 10.1111/j.1600-0447.2006.00924.x 17498156

[B135] Winston J. S. O’Doherty J. Kilner J. M. Perrett D. I. Dolan R. J. (2007). Brain systems for assessing facial attractiveness. *Neuropsychologia* 45 195–206. 10.1016/j.neuropsychologia.2006.05.009 16828125

[B136] Wise N. J. Frangos E. Komisaruk B. R. (2016). Activation of sensory and other brain regions in response to imagined versus physical genital stimulation. *Socioaffect. Neurosci. Psychol.* 6:631481. 10.3402/snp.v6.31481 PMC5084724 27791966

[B137] Wise N. J. Frangos E. Komisaruk B. R. (2017). Brain activity unique to orgasm in women: an fMRI analysis. *J. Sex. Med.* 14 1380–1391. 10.1016/j.jsxm.2017.08.014 28986148 PMC5675825

[B138] Xu A. Brown C. Feng W. (2011). Reward and motivation systems: a brain mapping study of early-stage intense romantic love in Chinese participants. *Hum. Brain Mapp.* 32 249–257. 10.1002/hbm.21017 21229613 PMC6870433

[B139] Xu L. Becker B. Luo R. Zheng X. Zhao W. Zhang Q. (2020). Oxytocin amplifies sex differences in human mate choice. *Psychoneuroendocrinology* 112:104483. 10.1016/j.psyneuen.2019.104483 31757429

[B140] Young K. A. Gobrogge K. L. Liu Y. Wang Z. (2011). The neurobiology of pair bonding: insights from a socially monogamous rodent. *Front. Neuroendocrinol.* 32:53–69. 10.1016/j.yfrne.2010.07.006 20688099 PMC3012750

[B141] Zeki S. (2007). The neurobiology of love. *FEBS Lett.* 581 2575–2579. 10.1016/j.febslet.2007.03.094 17531984

