# Supplementary material for: After the Honeymoon: Neural and Genetic Correlates of Romantic Love in Newlywed Marriages
Source: Front Psychol. 2020 May 7;11:634. doi: 10.3389/fpsyg.2020.00634 (PMC7223160; doi:10.3389/fpsyg.2020.00634)
Supplement: Supplementary file 1 [file Data_Sheet_1.PDF]

Supplementary Table 1. Regional brain activations correlated with changes in romantic love maintenance (T2-T1) across the first-year of newlywed marriages.

	Left					Right				
Brain Region	x	y	z	T	*p*	x	y	z	T	*p*
					Brain response at Time 1					
					ROI Activations					
VTA[1,2,3,4]						6	-12	-14	2.37	0.04
Raphe[1]						3	-27	-21	2.36	0.04
Pons[1]						0	-39	-45	3.09	0.04
Medial prefrontal cortex[4]						4	30	-9	3.09	0.04
Paracentral lobule[5]						6	-21`	57	2.27	0.05
					Whole-brain Activations					
Perirhinal cortex/fusiform gyrus						39	-18	-27	5.42	≤ .001
Superior frontal gyrus, anterior						15	54	39	4.73	≤.001
Superior frontal gyrus, medial						9	30	48	5.34	< .001
Superior temporal gyrus						48	-15	-3	5.22	≤ .001
Precuneus	-15	-42	42	4.36	≤ .001					
					Whole-brain Deactivations					
Inferior frontal gyrus						45	12	6	4.22	≤ .001
Middle frontal gyrus						33	27	27	4.21	≤ .001
Occipital cortex						9	-87	15	4.08	≤ .001
Collateral sulcus	-35	-48	0	4.83	≤ .001					

**B. Brain response at Time 2**										
ROI Activations										
Amygdala/Globus pallidus[1,3]						24	-3	-6	2.66	0.01
Mid-insula, posterior[1,3]	-44	-9	6	3.83	0.01					
Whole-brain Activations										
Occipital cortex, area 17	-1	-84	0	4.04	≤ .001	30	-57	6	6.27	≤ .001
Superior frontal gyrus						24	21	36	6.20	≤ .001
Superior temporal gyrus	-51	-12	9	4.39	≤ .001					
Parietal operculum/SII						48	-36	30	4.45	≤ .001
Precentral gyrus	-60	-12	30	4.48	≤ .001					
Supplementary motor area	-9	-6	57	7.42	≤ .001	12	-9	69	3.84	≤ .001
Whole-brain Deactivations										
Superior frontal gyrus, lateral						21	60	24	4.00	≤ .001
Occipital cortex	-15	-93	18	5.29	≤ .001					
Occipital cortex	-42	-84	9	4.01	≤ .001					

Note. All results are for regional brain activations significantly correlated with romantic love scores (T2-T1 Eros scores) across the first-year of newlywed marriages. Superscripts denote references for ROIs: [1] Acevedo et al. (2011); [2]Aron et al. (2005); [3] Bartels & Zeki (2004); [4]Xu et al. (2011).

Supplementary Table 2. Regional brain activations correlated with *AVPR1a rs3* (long alleles) and romantic love maintenance (T2-T1) across the first-year of newlywed marriages.

	Left					Right				
Brain Region	x	y	z	T	*p*	x	y	z	T	*p*
A. Brain response at Time 1										
ROI Activations										
Caudate tail[1]						33	-51	9	2.83	0.01
Septum/fornix[1,2]						0	0	18	2.26	0.03
Pons[1]						3	-39	-42	4.02	0.002
Amygdala/GP[1,3]						21	-3	-9	3.29	0.01
B. Brain response at Time 2										
ROI Activations										
VTA/SN [1]	-3	-18	-18	4.58	0.001					
Caudate, head[1]	-12	24	3	2.11	0.03					
Raphe nuclei[1]	-2	-27	-12	2.65	0.01	2	-21	-12	3.56	0.02
Hippocampus/caudate tail[1,2,4]	-33	-33	0	7.04	≤.001	36	-31	1	5.32	0.01
Posterior hippocampus[1]	-34	-34	-4	6.46	0.001	22	-34	6	3.41	0.01
Anterior cingulate cortex[1,3]	-12	42	18	5.33	0.001					
Occipital cortex[1]	-27	-60	15	4.12	0.01					
Whole-brain Activations										
Lateral geniculate						18	-21	-9	6.35	≤.001
Superior temporal gyrus						54	-39	18	4.67	≤.001

Note. All results are for regions showing greater brain activity associated with *AVPR1a rs3* (long alleles) and Eros scores (T2-T1). Superscripts denote references for ROIs: [1] Acevedo et al. (2011); [2]Aron et al. (2005); [3] Bartels & Zeki (2004); [4]Xu et al. (2011).

Supplementary Table 3. Regional brain activations correlated with *OXTR rs53576* (G alleles) and romantic love maintenance (T2-T1) across the first-year of newlywed marriages.

	Left					Right				
Brain Region	x	y	z	T	*p*	x	y	z	T	*p*
A. Brain response at Time 1										
ROI Activations										
VTA/SN[1]						3	-15	-21	4.43	0.01
Amygdala (lateral/basolateral)[4]						21	0	-24	6.07	≤ .001
Amygdala, central[1]	-30	-6	-24	3.61	0.01					
Hippocampus/entorhinal area[1]	-36	-3	-18	4.14	0.01	33	-3	-15	2.78	0.03
Whole-brain Activations										
Occipital/lingual gyrus	-3	-72	-6	6.73	≤ .001	15	-69	-9	7.60	≤ .001
Whole-brain Deactivations										
Superior frontal gyrus, mid	-9	51	48	4.72	≤ .001	12	15	60	6.69	≤ .001
Superior frontal gyrus, anterior	-18	63	21	6.46	≤ .001					
Middle frontal gyrus	-48	18	48	6.19	≤ .001					
B. Brain response at Time 2										
ROI Activations										
VP región, posterior[1,3]	-21	-3	-6	3.05	0.04					
Caudate[1,2]	-15	-9	21	2.66	0.05					
Amygdala, central[1]						27	-9	-12	3.00	0.03

					Whole-brain Activations					
Intraparietal sulcus, anterior						42	-42	42	5.41	≤ .001
Inferior frontal gyrus						54	21	12	4.35	≤ .001
Middle frontal gyrus						42	6	60	4.53	≤ .001
Superior temporal gyrus						51	-24	3	4.45	≤ .001
Dorsolateral prefrontal cortex	-48	42	21	5.82	≤ .001					
					Whole-brain Deactivations					
Caudate, anterior	-9	21	0	5.46	≤ .001					
Angular gyrus	-42	-84	30	6.54	≤ .001					
Somatosensory cortex	-24	-32	63	4.45	≤ .001					
Premotor/motor cortex	-6	-18	54	4.22	≤ .001	12	-15	69	5.94	≤ .001

Note. All results are for regions showing greater brain activity associated with *OXTR* rs53576 (G alleles) and Eros scores (T2-T1). Superscripts denote references for ROIs: [1] Acevedo et al. (2011); [2]Aron et al. (2005); [3] Bartels & Zeki (2004); [4]Xu et al. (2011).

Supplementary Table 4. Regional brain activations correlated with *DRD4* (7R alleles) and romantic love maintenance (T2-T1) across the first-year of newlywed marriages.

	Left					Right				
Brain Region	x	y	z	T	*p*	x	y	z	T	*p*
**A. Brain response at Time 1**										
ROI Activations										
Medial prefrontal cortex[4]	-3	30	-9	3.63	0.02	0	27	-9	3.58	0.02
Paracentral lobule[5]						6	-9	54	3.78	0.01
Whole-brain Activations										
Dorsolateral prefrontal cortex						-33	18	45	7.20	≤ .001
Entorhinal cortex						24	-18	-24	6.75	≤ .001
Primary somatosensory cortex	-39	-24	48	3.86	≤ .001					
Supramarginal gyrus/ Intraparietal sulcus	-48	-63	36	3.60	≤ .001					
Lateral prefrontal cortex	-27	54	6	5.07	≤ .001					
**B. Brain response at Time 2**										
Whole-brain Activations										
Secondary somatosensory cortex	-48	-9	6	6.32	≤ .001					
Dorsal prefrontal cortex	15	69	21	4.04	≤ .001					
Whole-brain Deactivations										
Hippocampus	-39	-24	-12	4.82	≤ .001	36	-24	-12	5.78	≤ .001
Temporal gyrus, anterior						30	21	-12	5.72	≤ .001

Note. All results are for regions showing greater brain activity associated with *DRD4* (7R alleles) and Eros scores (T2-T1). Superscripts denote references for ROIs: [1] Acevedo et al. (2011); [2]Aron et al. (2005); [3] Bartels & Zeki (2004); [4]Xu et al. (2011); [5] Wise et al., 2016.

Supplementary Table 5. Regional brain activations correlated with *COMT* rs4680 (A-alleles) and romantic love maintenance (T2-T1) across the first-year of newlywed marriages.

	Left					Right				
Brain Region	x	y	z	T	*p*	x	y	z	T	*p*
A. Brain response at Time 1										
ROI Activations										
Medial prefrontal cortex[4]	-3	30	-9	3.49	0.01	0	24	-9	5.08	0.01
Whole-brain Activations										
Primary somatosensory cortex						63	-15	42	7.37	$\leq$ .001
Secondary somatosensory cortex	-57	0	12	6.17	$\leq$ .001					
Whole-brain Deactivations										
Hippocampus						24	-30	-9	4.07	$\leq$ .001
B. Brain response at Time 2										
ROI Activations										
Paracentral lobule[5]	-3	-24	57	3.13	0.02					
Whole-brain Activations										
Ventrolateral prefrontal cortex						42	54	-9	6.11	$\leq$ .001
Dorsolateral prefrontal cortex						48	15	51	6.02	$\leq$ .001
Posterior cingulate cortex						6	-39	36	5.03	$\leq$ .001
Whole-brain Deactivations										
Substantia nigra						15	-15	-15	4.78	$\leq$ .001
Caudate tail						24	-36	9	5.57	$\leq$ .001

Dorsal midbrain	3	-24	-12	4.30	$\leq$ .001

Note. All results are for regions showing greater brain activity associated with *COMT* rs4640 (A-alleles) and Eros scores (T2-T1). Superscripts denote references for ROIs: [1] Acevedo et al. (2011); [2]Aron et al. (2005); [3] Bartels & Zeki (2004); [4]Xu et al. (2011); [5] Wise et al., 2016.
